# Supplementary material for: Down-selection of biomolecules to assemble “reverse micelle” with perovskites
Source: Nat Commun. 2024 Jan 26;15:772. doi: 10.1038/s41467-024-44881-4 (PMC10817902; doi:10.1038/s41467-024-44881-4)
Supplement: Supplementary file 1 — Supplementary Information [file 41467_2024_44881_MOESM1_ESM.pdf]

Supplementary Information

for

Down-Selection of Biomolecules to Assemble "Reverse Micelle" with Perovskites

Haodong Wu,[#] Yuchen Hou,[#] Jungjin Yoon, Abbey Marie Knoepfel, Luyao Zheng, Dong Yang, Ke Wang, Jin Qian, Shashank Priya[*], Kai Wang[*]

[#] These authors contributed equally
* Corresponding authors: sup103@psu.edu and kaiwang@psu.edu

This file includes:

Supplementary Note 1 to Note 5

Supplementary Figures 1 to 29

Supplementary Tables 1 to 7

Supplementary References

Table of Contents

Supplementary Notes 3

Supplementary Note 1 Fermi level calculation from KPFM 3

Supplementary Note 2 Electron Schottky barrier analysis at perovskite/HTL interface .. 4

Supplementary Note 3 Recombination study in solar cell device 5

Supplementary Note 4 Literature survey of corresponding perovskite solar cells/modules 8

Supplementary Note 5 Formation mechanism of "reverse micelle" and "bilayer" 9

Supplementary Figures 3-29 12

Supplementary Tables 1-7 47

Supplementary References 54

Supplementary Notes

Supplementary Note 1 Fermi level calculation from KPFM

From the results of KPFM characterization, the Fermi level of perovskite film can be obtained by calculating the difference between the work function of sample and the KPFM tip, which is called the CPD:[1]

$$\mathrm{CPD} = \frac{\Phi_{tip} - \Phi_{sample}}{e} \qquad \text{Supplementary Equation (1)}$$

In which *e* refers to the elementary charge, Φ_{sample} is the work function of the sample surface, and Φ_{tip} is the work function of the tip. Cause the results of KPFM characterization are in the form of CPD, a reference material called HOPG with a work function of 4.6 eV is needed to calculate the work function of the tip,[2] $\Phi_{tip} = CPD_{HOPG} + 4.6$ eV. Based on this, the Fermi level of the perovskite film ($E_{F,\ sample}$) can be obtained by:[1]

$$E_{F,\ sample} = e\left[-4.6+(CPD_{sample} - CPD_{HOPG})\right] \qquad \text{Supplementary Equation (2)}$$

Where CPD_{sample} and CPD_{HOPG} are the *CPD* measured on perovskite film surface and reference HOPG film surface. The CPD mapping of HOPG, camphor-perovskite, pristine $MAPbI_3$ surface can be found in Supplementary Figs. 17a-c. The CPD histogram spectra of HOPG, pristine $MAPbI_3$ and camphor-$MAPbI_3$ film can be found in Supplementary Fig. 17d, in which CPD_{HOPG} was measured to be ~705 mV, CPD_{MAPbI3} was measured to be ~647 mV and $CPD_{Camphor\text{-}doped\ MAPbI3}$ was measured to be ~979 mV. Therefore, the Fermi level of pristine $MAPbI_3$ and camphor-$MAPbI_3$ can be calculated as -4.66 eV and -4.32 eV, as shown in Supplementary Fig. 17e.

Supplementary Note 2 Electron Schottky barrier analysis at perovskite/HTL interface

Spiro-OMeTAD with large amounts of ionic dopants like Li-TFSI is typically used as HTL in the perovskite solar cells.[3] Due to the ionic dopants, the Fermi level of spiro-OMeTAD moves towards its valence band maximum (VBM) significantly, leading its energy level diagram acting like that of metal materials. Supplementary Fig. 18a shows the scheme of energy level of perovskite layer and spiro-OMeTAD layer before contact under dark condition, in which the spiro-OMeTAD HTL has a much lower Fermi level. Upon contact as shown in Supplementary Fig. 18b, the electrons of the perovskite layer will diffuse into the HTL and the holes will remain in the perovskite layer, leading to the formation of depletion region. This depletion region will induce an internal electric filed (build-in potential) which retard the diffusion of electrons from perovskite layer to HTL, resulting in the bending up effect of conduction band minimum (CBM) and VBM of the perovskite layer at the contact interface. In this way, the Schottky barrier is formed and its barrier height Φ_B can be calculated:[4]

$$\Phi_B = \Phi_{HTL} - \Phi_{pero} \qquad \text{Supplementary Equation (3)}$$

where Φ_{HTL} and Φ_{pero} are the work function of HTL and perovskite, respectively. The Schottky barrier can effectively inhibit the diffusion of electrons from the perovskite layer to HTL as well as the suppress the interfacial recombination, and the energy level bending effect can enhance the hole transportation across the perovskite/HTL interface. Based on this, the camphor-perovskite layer with higher Fermi level compared to that of the pristine $MAPbI_3$ will have larger Schottky barrier, which is supposed to provide a more efficient electron blocking effect at the anode and thus less recombination losses. Under light condition, the situation is similar, the only difference is that the Fermi level E_F of the perovskite layer will spill into two levels $E_{F, e}$, $E_{F, h}$ near CBM and VBM. Just as discussed before, the VBM of perovskite layer forms ohmic contact with the HTL, and the CBM of perovskite layer forms Schottky barrier with HTL, $\Phi_{B, e}$, which can be calculated:

$$\Phi_{B, e} = \Phi_{HTL} - \Phi_{p, e} \qquad \text{Supplementary Equation (4)}$$

where Φ_{HTL} and $\Phi_{p, e}$ are the work function of HTL and the value of Fermi level near the CBM of perovskite layer, respectively.

Supplementary Note 3 Recombination study in solar cell device

To verify the reduced losses of recombination, we further investigate the recombination process of different solar cell devices in Fig. 4a. The overall improvement of V_{OC} is graphically exemplified in Fig. 4a(i), by comparing the light current density-voltage (J-V) curve between pristine and camphor-$MAPbI_3$ devices. There is a simultaneously enhanced V_{OC} (*ca.* 10% enhancement) and FF from pristine to the biomolecule incorporated device. The FF is in principle related to the charge transfer characteristics throughout the device which can be more visibly revealed at smaller internal bias region. This information can be reflected from the quantification of the photocurrent ($J_{ph} = J_i - J_d$) as a function of effective bias ($V_{eff} = V_0 - V$) (Fig. 4a(ii)), where J_i is the current density under illumination, J_d is the dark current, V_0 is the voltage when $J_{ph}= 0$, and V is the applied bias voltage, respectively.[5] J_{ph} from both curves are saturated to almost identical value of J_{sat} under large V_{eff} (> 0.8 V), indicating under such large internal field intensity, the population of collected photocarriers are identical for both devices. While at lower V_{eff} region (< 0.15 V), the camphor-$MAPbI_3$ device always exhibit higher J_{ph} than the pristine device. This indicates even with such a lower driving force, the photocarriers from camphor-$MAPbI_3$ device can still be more efficiently collected, which can be a result of the better electron blocking effect at the perovskite/HTL interface due to the higher Fermi level of the top surface of camphor-$MAPbI_3$ film (Fig. 4a(iv)). To verify the hypothetically reduced recombination losses, we also performed the light intensity-dependent V_{OC} and J_{SC} investigation (Figs. 4a(iii) and 4a(iv), respectively). Fundamentally, under open-circuit conditions, V_{OC} can be correlated with the illumination intensity by[5]

$$V_{OC} \propto S \cdot \ln(I) \qquad \text{Supplementary Equation (5)}$$

where S is the slope and I is the light intensity, respectively. Near the strong light intensity (e.g., one-sun illumination here), in an ideal case where no traps are present in the device, the recombination mechanism is only dominated by the bimolecular recombination (or radiative recombination). Under this condition, using a classic photodiode equation, the slope can be quantified by $S_0 = kT/e$, with k being the Boltzmann constant, T the absolute temperature, and e is the elementary charge, respectively. In real cases, there are inevitable traps present throughout the device, and the deviation of the slope from S_0 can be ascribed to another type of recombination, i.e., the trap-assisted recombination[6] (Shockley-Read-Hall, SRH recombination). Fig. 4a(iii) shows the pristine device having a more deviated slope of 1.95 kT/e than that of camphor-$MAPbI_3$

device (1.72 kT/e) from the ideal case (1 kT/e), suggesting reduced SRH recombination losses in the camphor-$MAPbI_3$ device. Similarly, we investigate the J_{SC} dependence on light intensity, which theoretically follows the relationship of [7]

$$J_{SC} \propto I^{\alpha} \qquad (\alpha \leq 1) \qquad \text{Supplementary Equation (6)}$$

where α is an index. In an ideal case, under the short-circuit condition, the bimolecular recombination would be minimized due to the maximal sweep out of carriers, consequently leading to a linear correlation between current and light intensity, i.e., $\alpha = 1$. In other words, deviation of α from 1 can be a result of insufficient charge sweep out induced by recombination losses. Fig. 4a(iv) shows the power-law fitting of the log-log plot deviated from Supplementary Equation 6, where there is a larger deviation from the pristine device (smaller $\alpha = 0.88$) compared to the camphor-$MAPbI_3$ device ($\alpha = 0.94$). These results reveal that the presence of the top layer of camphor-$MAPbI_3$ triggers a stronger electron blocking effect thereby minimizing the recombination losses.

The beneficial role of the higher Fermi level from the perspective of electrical recombination losses in the device has been discussed thus far. Here we also investigate how such a self-assembled nanoparticle layer would positively contribute to the higher potential energy of the photocarriers. Photoluminescence (PL) and electroluminescence (EL) investigations were both carried out on the samples. Figs. 4b(i) and 4b(ii) schematize the difference in the mechanisms between PL and EL, respectively. Briefly, by constructing an interface of perovskite/HTL, the photo-excited electrons have three potential pathways, i.e., ❶ SRH recombination losses; ❷ PL emission; and ❸ diffusion to HTL (Fig. 4b(i)), with a correlation of:

$$n_{ex} = n_{SRH} + n_{PL} + n_{dif} \qquad \text{Supplementary Equation (7)}$$

where n_{ex} is the total population of photoexcited electrons, n_{SRH}, n_{PL}, and n_{dif} are the population of the excited electrons proceed towards SRH recombination (❶), PL emission (❷), and diffusion to HTL (❸), respectively. It should be noted that simply the result of n_{PL} from PL measurement cannot reveal the ratio between SRH losses and diffusion to HTL, where the former is harmful to device performance but the latter is beneficial. In real device working conditions, due to the presence of internal field (asymmetric electrodes), the charge transfer across the perovskite/HTL interface is dominated by a drifting process rather than a simple diffusion, which will make the above model less applicable for analyzing these real cases. Thus, additional information is needed to decipher competition between SRH losses and diffusion in order to judge which device works

better. One solution to this issue is the EL (electroluminescence) study using the solar cell device but reverse the charge flow by current injection. As shown in Fig. 4b(ii), the same solar cell devices are utilized, and an identical current (population of electrons) is injected into the devices with the same potential energy, and the consumption of these injected electrons can also have three pathways, i.e., ① SRH recombination losses; ② electroluminescence (bimolecular recombination) emission; and ③ loss during charge transfer (CT) across multiple interfaces and within layers. However, both ① and ③ are harmful to the device which is supposed to be minimized in order to maximize the device performance. Compared to the PL, the result from EL can be a direct index to evaluate the device performance, as the higher EL (②) intensity the less losses via either ① or ③ or both. We carry out both PL and EL investigations for devices using either pristine or camphor-$MAPbI_3$ perovskite. Fig. 4c(i) shows the PL results of samples consisting of perovskite with a top Spiro-OMeTAD HTL. The pristine sample displays a spectrum with a peak around 1.58 eV which is consistent to the bandgap of $MAPbI_3$. In contrast, the camphor-$MAPbI_3$ perovskite displays a blue shift (Supplementary Fig. 20b(ii)), exhibiting a peak at 1.63 eV, accompanied by lower states peaked at 1.58 eV. The larger bandgap of 1.63 eV is most likely belong to the particle-like perovskites in the upper layer (Supplementary Fig. 14c) while the lower states (1.58 eV) are consistent to the bottom columnar grains identical to the pristine. As mentioned above, albeit the camphor-$MAPbI_3$ perovskite displays lower PL intensity, it is difficult to conclude whether the lower radiative intensity comes from augment in ❶ or ❸. Similarly, transient PL (Supplementary Fig. 20b(iii)) with information of photocarrier lifetime also cannot distinguish either contribution. Instead, the EL result could give a more convincing conclusion. As shown in Fig. 4c(ii), the camphor-$MAPbI_3$ device exhibits a five-fold higher EL intensity than the pristine one, suggesting reduced losses through ① or ③ or both, which is consistent with the results of recombination studies. Similarly, two sub-peaks were observed which are centered at 1.58 and 1.63 eV, respectively, from the camphor-$MAPbI_3$ device (Supplementary Fig. 20c(iv)). This is consistent to the PL results and suggests that besides the conduction band frontier states (corresponding to 1.58 eV), there is an additional excited state of electrons at an energy level 50 meV higher (corresponding to 1.63 eV) (Supplementary Fig. 20c(v)). The higher energy state can also contribute to a higher potential energy to the excited electrons, jointly with the reduced recombination losses, leading to the higher V_{OC} of the devices with Tier 2 class biomolecules.

Supplementary Note 4 Literature survey of corresponding perovskite solar cells/modules

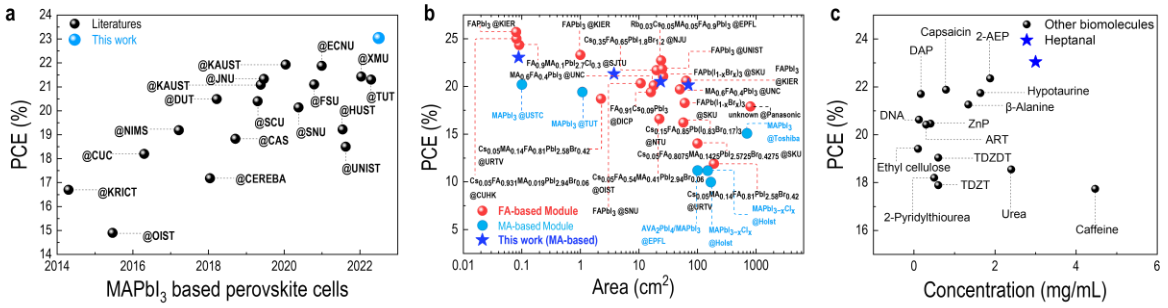

Supplementary Fig. 1. PCE summary of corresponding perovskite solar cells/modules in literatures and this work.

a PCE survey plot of selected $MAPbI_3$ based perovskite solar cells since 2014 along with affiliations. (Obtained from references: [5,8-24])

b PCE vs. device active area survey plot of perovskite solar cells along with compositions and affiliations. (Obtained from references: [16,25-41])

c PCE survey plot of perovskite solar cells with different biomolecule additives with different doping concentrations. (Obtained from references: [22,42-51])

Supplementary Note 5 Formation mechanism of "reverse micelle" and "bilayer"

Reverse micelle: A "Micelle" is an aggregate (or supramolecular assembly) consisting of surfactant molecules (e.g., phospholipid molecules typically in bio-cell membrane). They form associated colloidal system in a typical case of surfactants and water system.[52] The functional molecules share the similarity of a hydrophilic "head" and hydrophobic "tail", as shown in Supplementary Figs. 2a-c. And in aqueous condition, the hydrophilic "head" tends to contact with the polar water solvent, while the hydrophobic "tail" attracts each other. This can lead to certain microstructures, including micelle models (head facing outside and tails are oriented toward the center of the sphere), lamellar models, and rod models. These various manifestations are driven by the combination of different molecular interactions present in the system, mainly including the (i) tail-tail interaction and (ii) head-solvent attraction.[53]

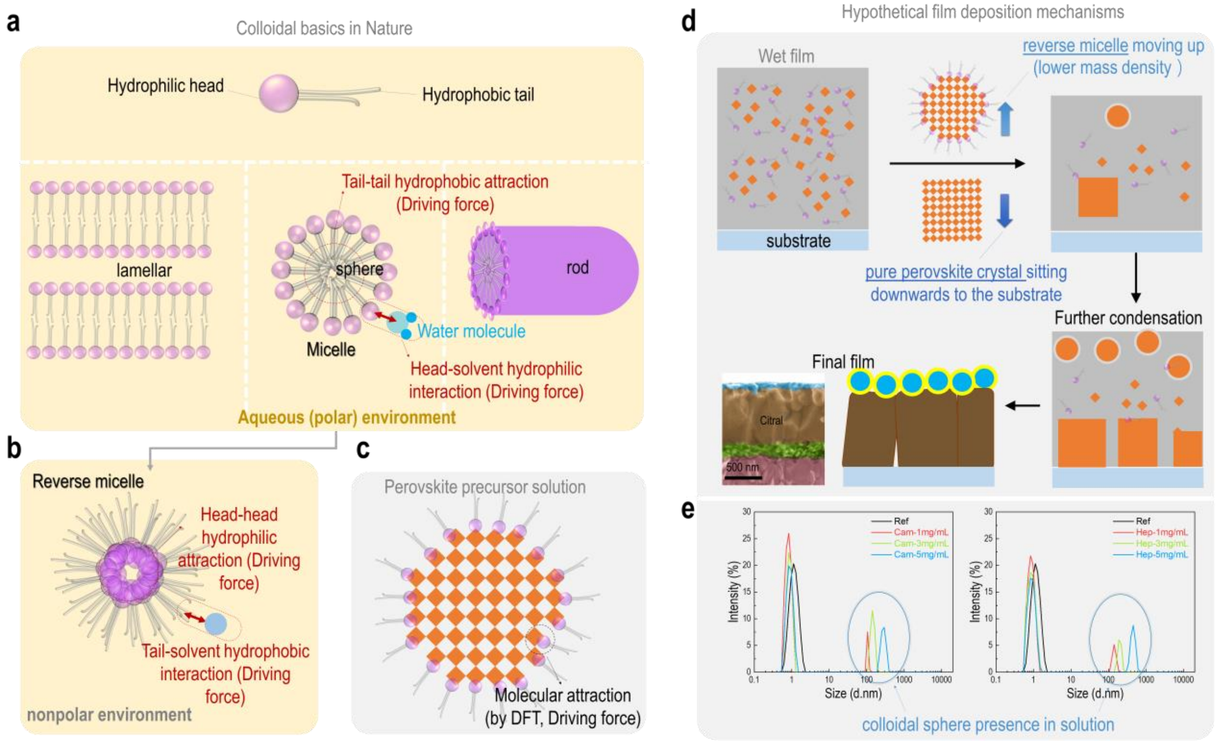

Supplementary Fig. 2. Illustration of formation mechanism of "reverse micelle" and "bilayer". a-c Hypothetical reverse micelle formation mechanisms, **a** colloidal basics in nature including amphiphilic molecule, lamellar, micelle (polar environment) and rod structures, **b** scheme of reverse micelle structure in nonpolar environment, **c** hypothetical perovskite-biomolecule reverse micelle structure. **d-e** Hypothetical film deposition mechanisms, **d** scheme of processes of bilayer formation, **e** dynamic light scattering spectra of corresponding perovskite

precursor solutions indicating the colloidal sphere (perovskite-biomolecule reverse micelle) presence in solution.

In our case the solution systems incorporate both these amphiphilic molecules and the perovskite precursors, leading to complex ingredients. Particularly the presence of the interaction between the perovskite and the additive molecules (Pb-“head” interaction) triggers the formation of a “reverse micelle” structure. We tested the dynamic light scattering (DLS) measurement on different samples and observed a secondary peak at a larger size region (appeared at a size level of 100s nm), in distinct contrast to the pristine samples that only exhibit a mono-peak at 1s nm level, as shown in Supplementary Fig. 2e.

This formation driving force can be understood by the molecular interaction between the carbonyl head and the perovskite lattice thoroughly present during the wet film drying process. This molecular attraction will lead to the biomolecules adsorbed onto the perovskite surface, with the hydrophobic tail of the biomolecules facing outside, which is also the reason for the higher hydrophobicity of the film (as verified by contact angle and water dropping tests in this study). However, formation of such a “reverse micelle” is not easily accessible and requires several conditions. We have shown there are five possible pathways leading to different mechanisms in Supplementary Fig. 16f. Not all the additive biomolecules can give rise to such a “reverse micelle” structure. In this study, we extensively investigate this unexpected phenomenon through a down-selective screening methodology.

Bi-layer: In regard to the bi-layer formation from the molecular self-assembly, we also present the possible mechanisms as shown in Supplementary Fig. 2d. In the wet film, both biomolecules and perovskite precursors are present in the system. Along with the solvent evaporation, the system is concentrating and the perovskite starts to nucleates and grow. Because of the small amount of the biomolecules added in this work, there are two manifestations of perovskite crystals in the drying wet film: (i) reverse micelle nanocrystal where biomolecules are adsorbed onto the surface of perovskite nanocrystals; (ii) regular pristine perovskite crystals where no biomolecules are adsorbed and the crystallization follows its original manner to condense into large grain pristine crystals. Because of the smaller mass density of the reverse micelle crystals than the pristine ones, they tend to float upwards, leaving the pristine crystal sitting at the bottom. It should be noted that there can also be other chemical and dynamical reasons that the reverse micelle particles move to

the top surface. For example, surface tension effect, and solvent steam dragging effect (solvent may interact with the outside biomolecules of the reverse micelle particle and drag it to the top when the solvent steam moving upwards) can also be possible, which however is beyond the scope of biomolecule down selection of this work but could lead to many interesting branches for future study. Some hints for future investigations: in classic colloidal scenario, modulation of the size and shapes into e.g., ellipsoids, cylinders, and bilayers are also possible during the micellization, as the shape and size of a micelle can be a function of the molecular geometry of its surfactant molecules as well as the solution conditions such as temperature, pH, surfactant concentration, and ionic strength, etc. Changing the conditions and biomolecules of other functional groups may chemically trigger new material discovery throughout the molecular level (e.g., organic-inorganic superlattice) to mesoscopic levels (e.g., perovskite-biomolecule framework).

Supplementary Figures 3-29

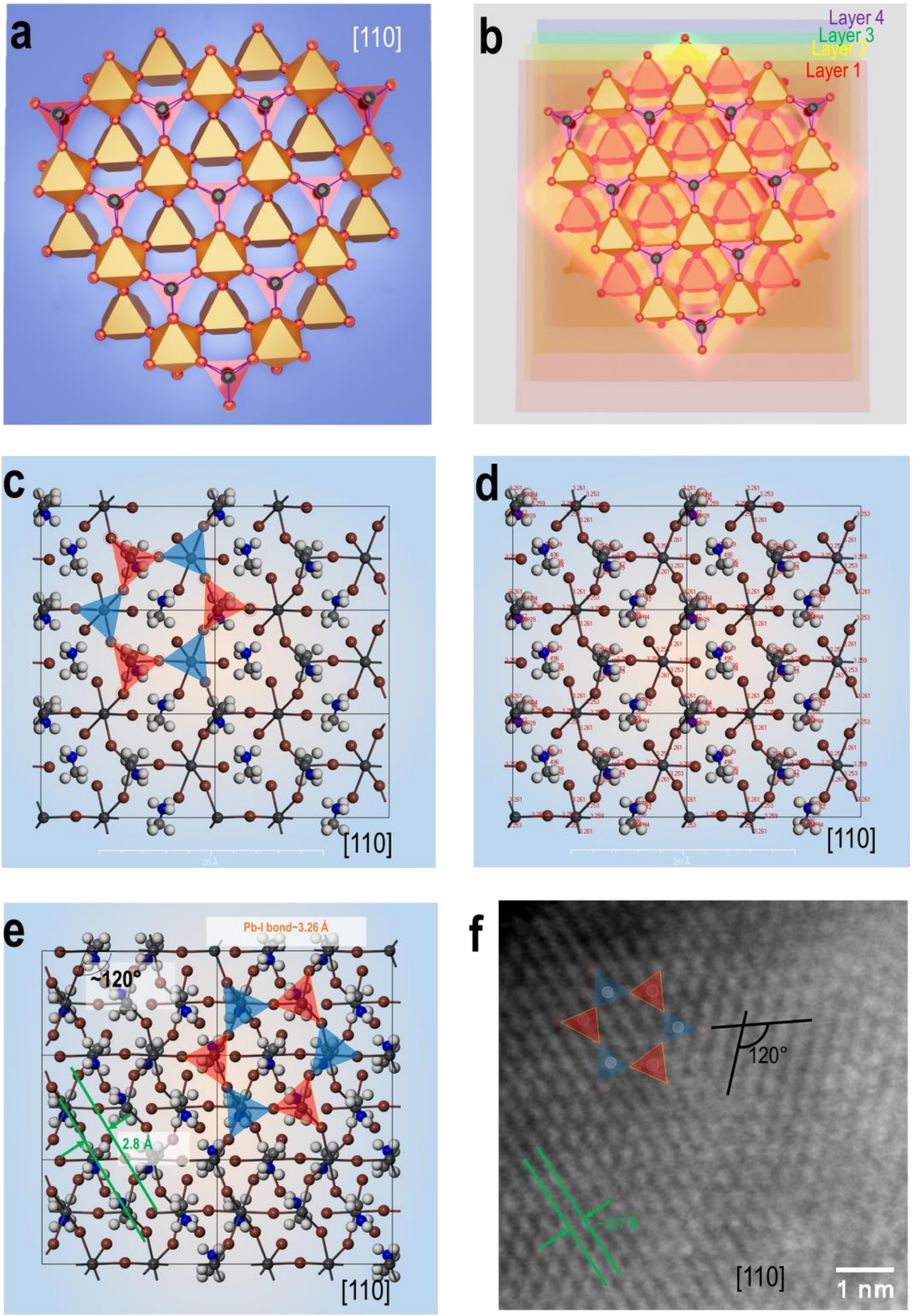

Supplementary Fig. 3. Schematical demonstration of $MAPbI_3$ perovskite crystal lattice structure. a The prototype perovskite of $MAPbI_3$ crystal with an exposure along the [110] direction. **b** Scheme showing different plane layers along the [110] direction. **c** Ball-stick manifest

of the lattice along the [110] direction. The blue and red triangle reveals the iodine at the sample planes (topmost plane). **d** Ball-stick manifest of the lattice showing the bond length values with a unit of Å. **e** Ball-stick manifest showing the plane distance and Pb-I bond length, which is corresponding to the results in the HRTEM image in **f**.

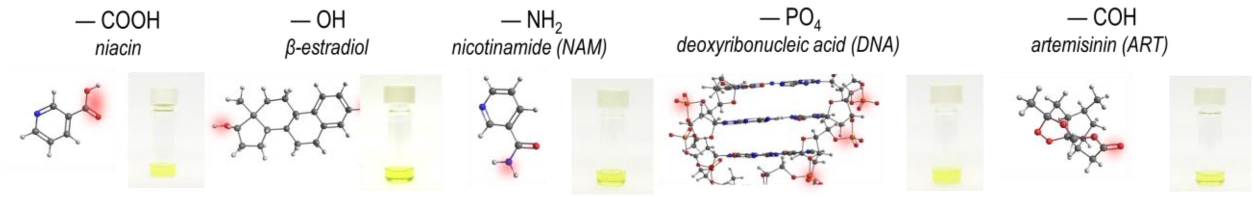

Supplementary Fig. 4. The molecular structure of Tier 1 class general biomolecules. Sequentially, niacin, β-estradiol, nicotinamide, deoxyribonucleic acid, artemisinin (gray, white, blue, red balls indicate C, H, N, O respectively) on the left side. Photography (right side of each group) of corresponding perovskite solutions.

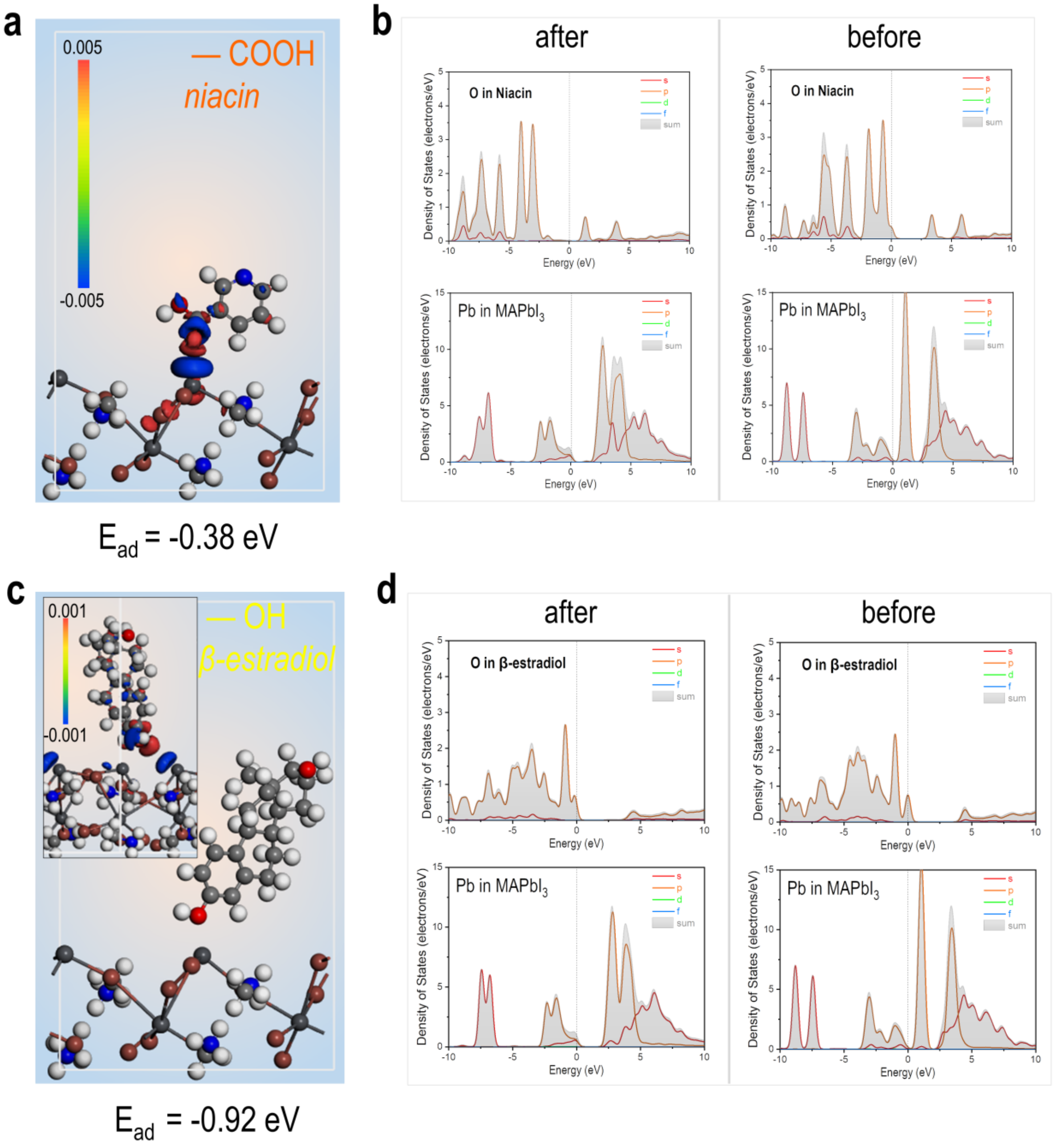

Supplementary Fig. 5. Computational illustration for interaction between perovskite and Tier 1 biomolecules. Density function theory (DFT) simulation of interactions between $MAPbI_3$ and **a** niacin, **c** β-estradiol molecule. Density of States (DOS) of O atom of **b** the carboxyl group in the niacin, **d** the hydroxyl group in the β-estradiol molecule and Pb atom in the $MAPbI_3$ lattice, before and after adsorption.

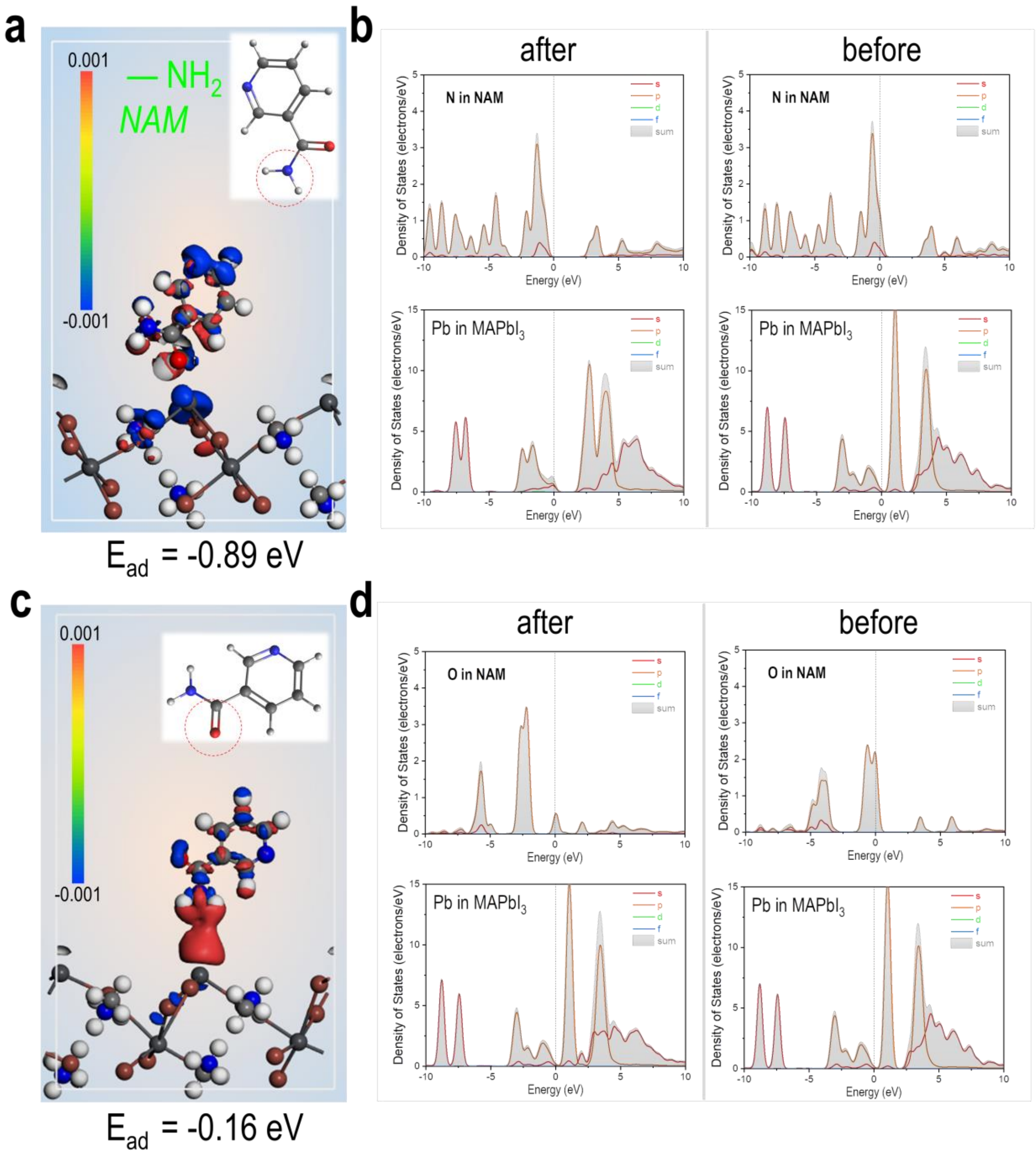

Supplementary Fig. 6. Computational illustration for interaction between perovskite and Tier 1 biomolecules. DFT simulation of interactions between $MAPbI_3$ and **a** amino group, **c** carbonyl group of nicotinamide. DOS of **b** N atom of the amino group, **d** O atom of the carbonyl group in the nicotinamide and Pb atom in the $MAPbI_3$ lattice, before and after adsorption.

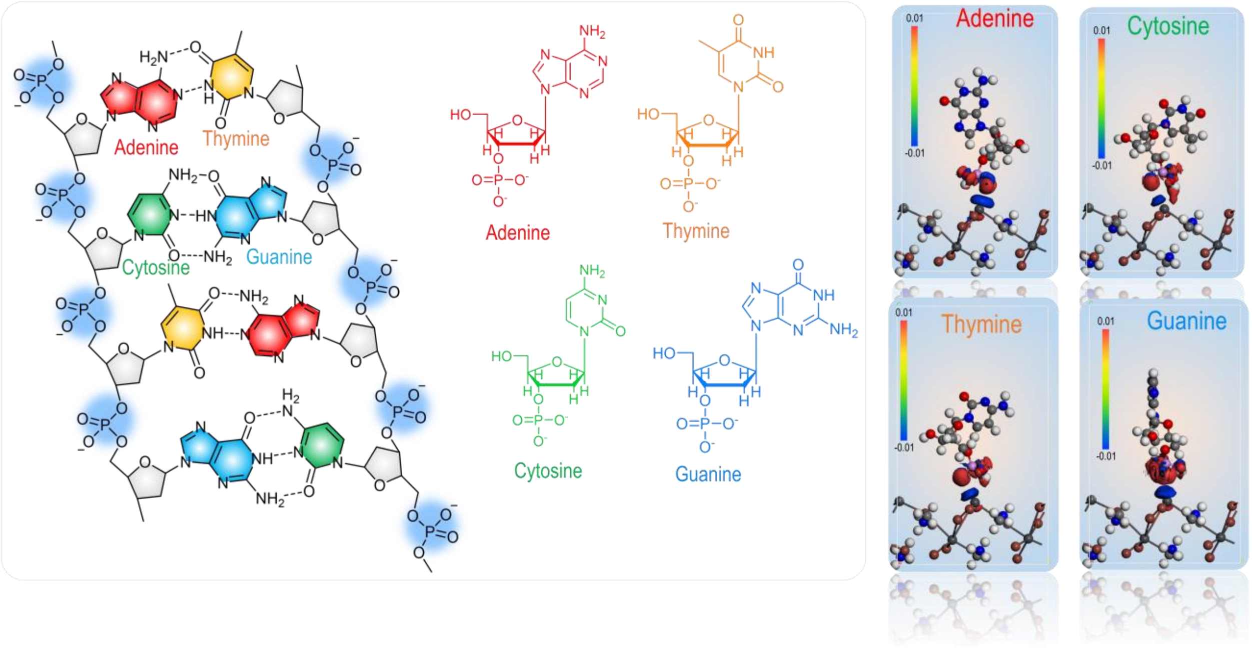

Supplementary Fig. 7. The molecular structure of DNA. In which there are two purines (adenine, thymine) and two pyrimidines (cytosine, guanine) on (left panel). DFT model of absorption of between purines, pyrimidines and perovskite along [110] direction on the perovskite lattice (right panel).

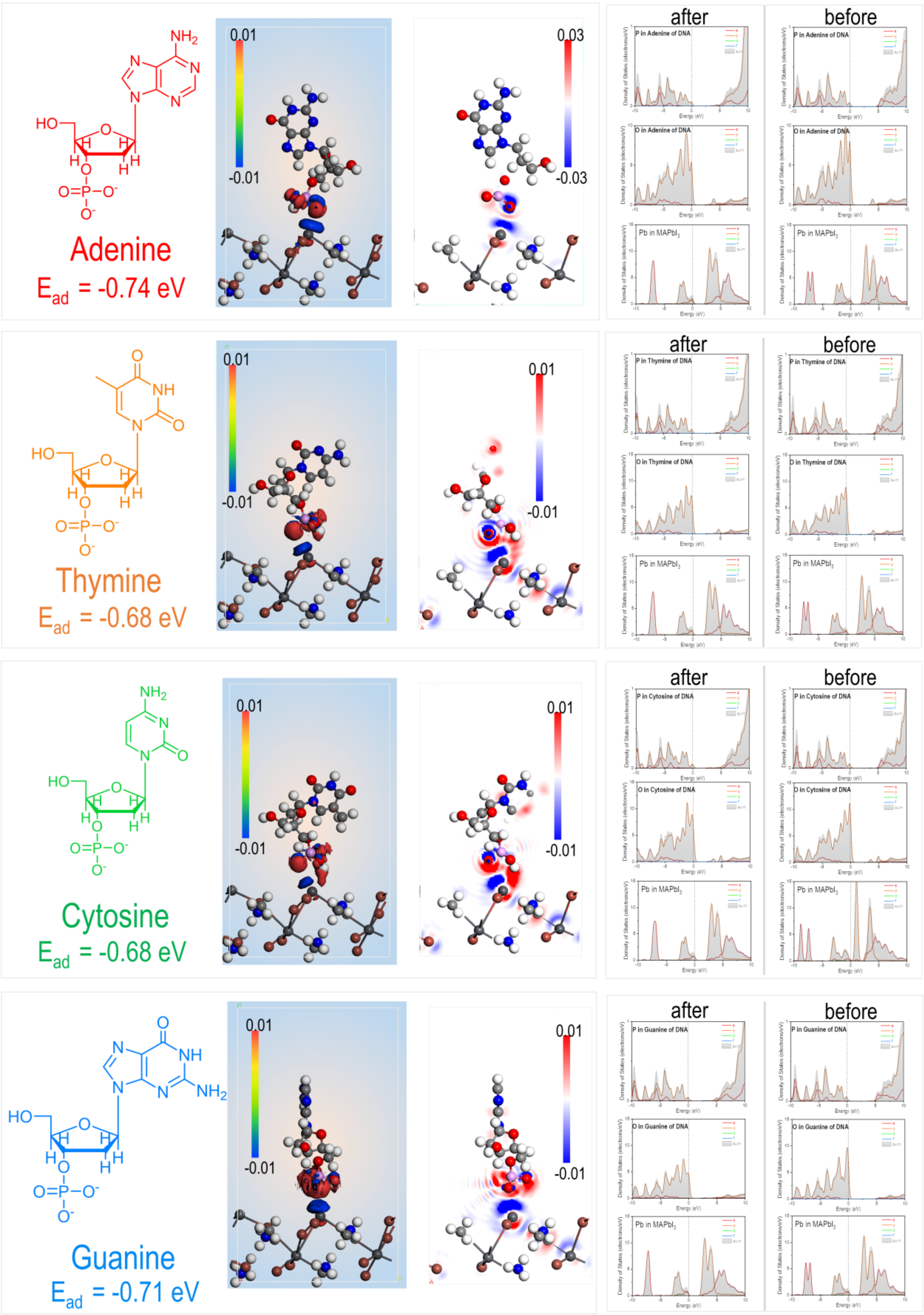

Adenine
E_{ad} = -0.74 eV
Thymine
E_{ad} = -0.68 eV
Cytosine
E_{ad} = -0.68 eV
Guanine
E_{ad} = -0.71 eV
after
before
P in Adenine of DNA
O in Adenine of DNA
Pb in $MAPbI_3$
P in Thymine of DNA
O in Thymine of DNA
P in Cytosine of DNA
O in Cytosine of DNA
P in Guanine of DNA
O in Guanine of DNA
0.01
-0.01
0.03
-0.03

Supplementary Fig. 8. Computational illustration for interaction between perovskite and Tier 1 biomolecules. Detailed DFT simulation of interactions between corresponding purines, pyrimidines and perovskite at along [110] direction and DOS of P atom, O atom of the phosphate group in the corresponding purines, pyrimidines and Pb atom in the $MAPbI_3$ lattice, before and after adsorption.

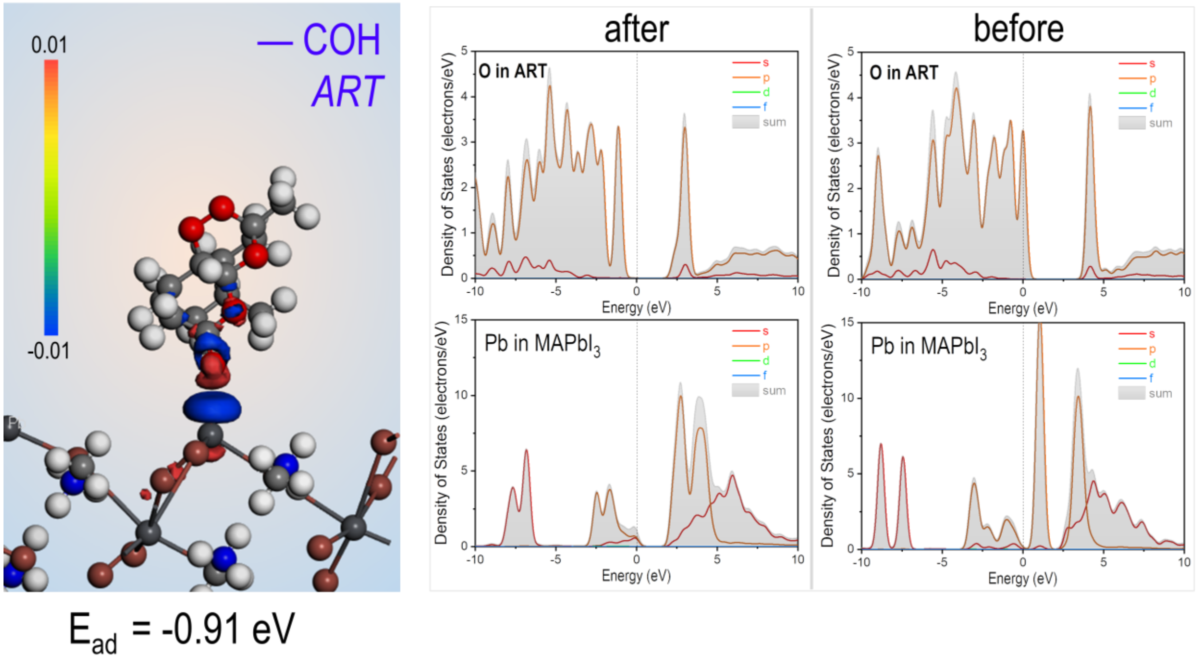

Supplementary Fig. 9. Computational illustration for interaction between perovskite and Tier 1 biomolecules. DFT simulation of interactions between artemisinin and perovskite along [110] direction and DOS of O atom of the carbonyl group in the artemisinin and Pb atom in the $MAPbI_3$ lattice, before and after adsorption.

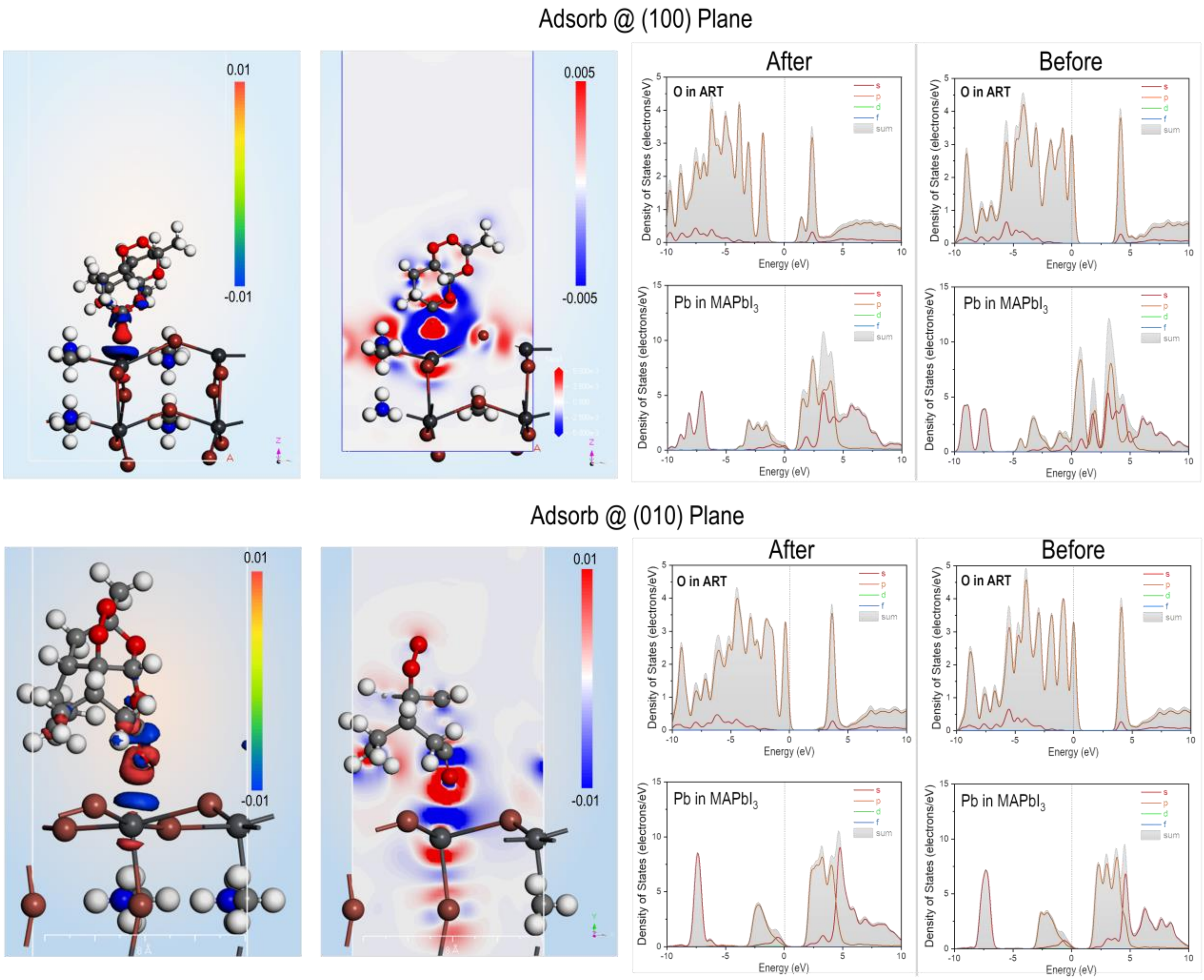

Supplementary Fig. 10. Computational illustration for interaction between perovskite and Tier 1 biomolecules. DFT simulation of interactions between artemisinin and perovskite and DOS of O atom of the carbonyl group in the artemisinin and Pb atom in the $MAPbI_3$ lattice, before and after adsorption at (100) and (010) planes.

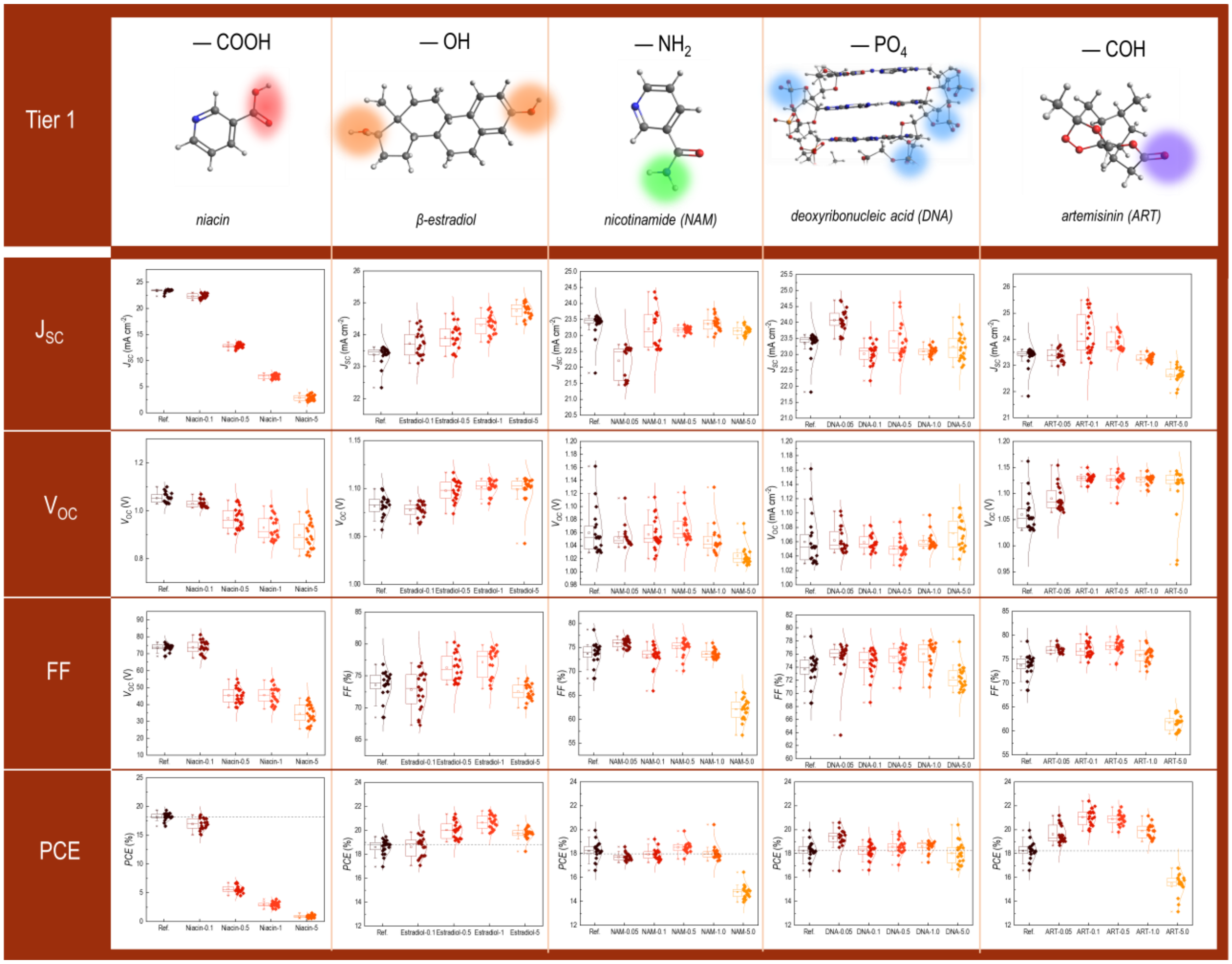

Supplementary Fig. 11. Statistic data of photovoltaic parameters of perovskite solar cells with Tier 1 general biomolecules. Including niacin, β-estradiol, nicotinamide, deoxyribonucleic acid, artemisinin with doping concentrations of 0, 0.05, 0.1, 1.0, 5.0 mg mL^{-1} (20 devices for each group).

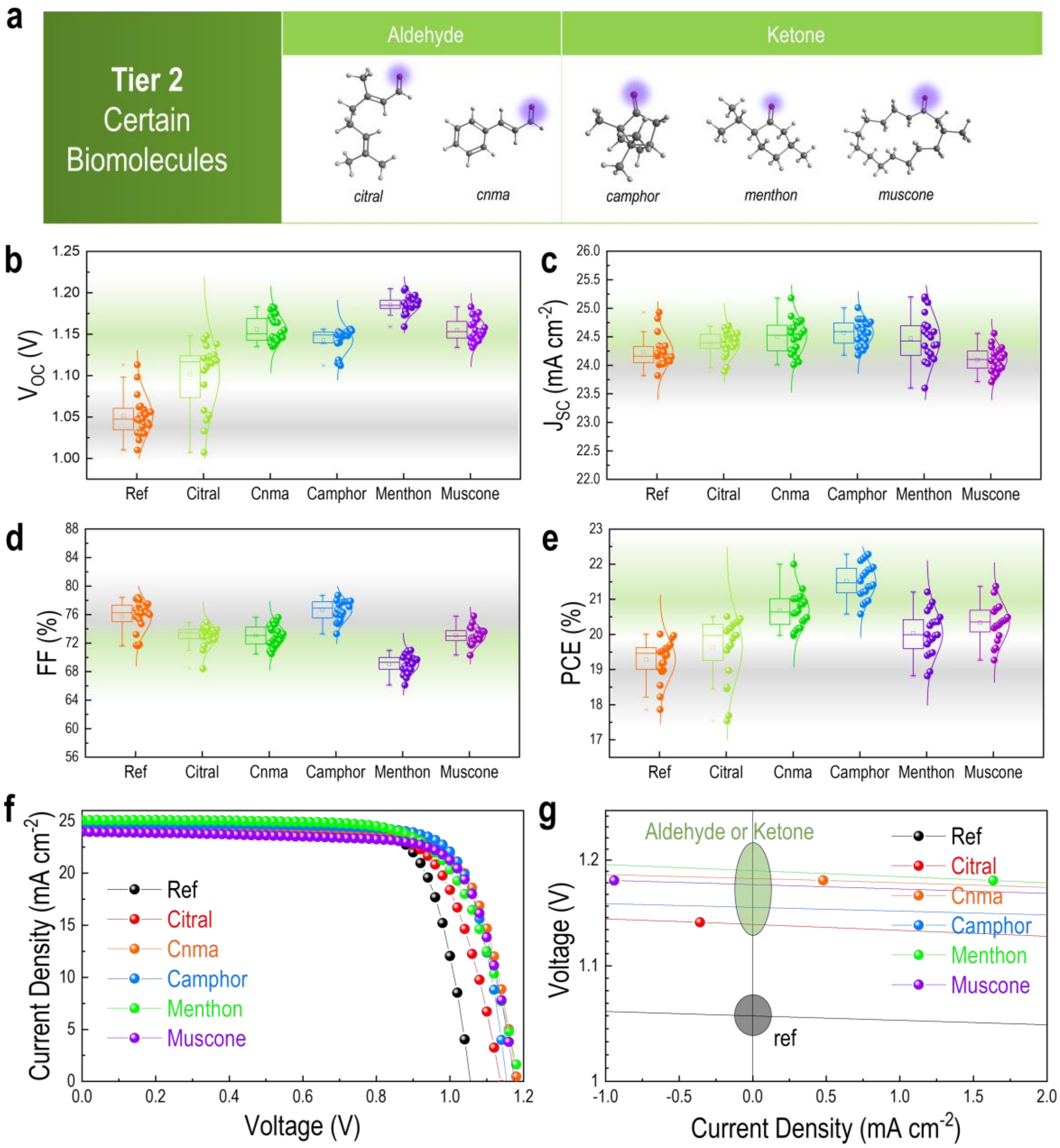

Supplementary Fig. 12. Device performance for perovskite solar cells with Tier 2 biomolecules. a Molecule structure of Tier 2 certain biomolecules, including citral, cinnamaldehyde, camphor, menthon, muscone. **b-e** Statistic data of photovoltaic parameters of perovskite solar cells with Tier 2 certain biomolecules of 3.0 mg mL^{-1} doping concentration. **f** J-V characteristic of perovskite solar cells with corresponding biomolecules. **g** Enlarged V-J characteristic at current density of -1 to 2.0 mA cm^{-2} regions, of different solar cells with corresponding biomolecules.

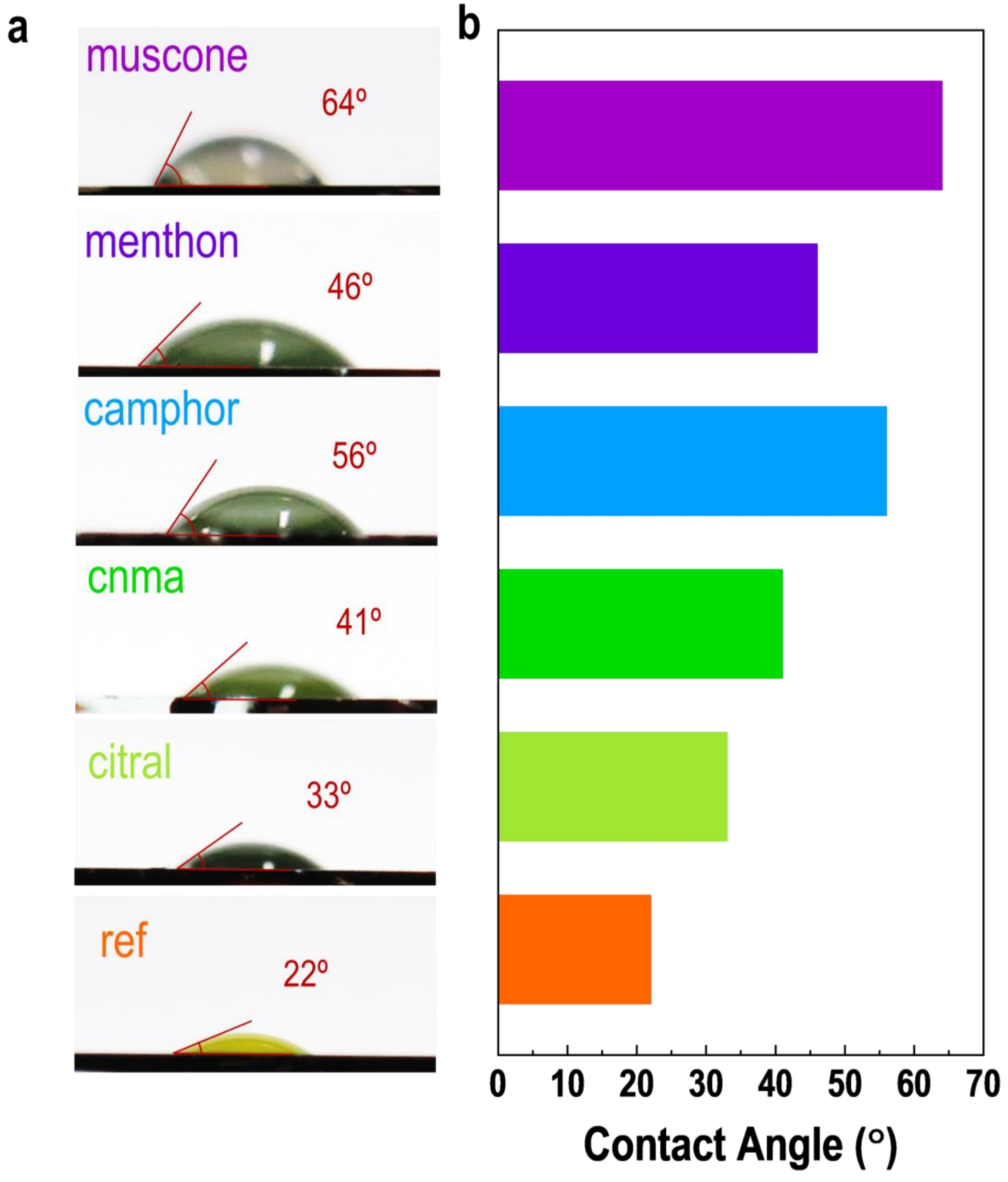

Supplementary Fig. 13. Hydrophobicity test for perovskite thin films with Tier 2 biomolecules. a Contact angle measurement of perovskite films with corresponding biomolecules by dropping one water droplet (50 μL) onto the surface. **b** Column plot of the contact angles horizontally aligned with corresponding perovskite films in **a**.

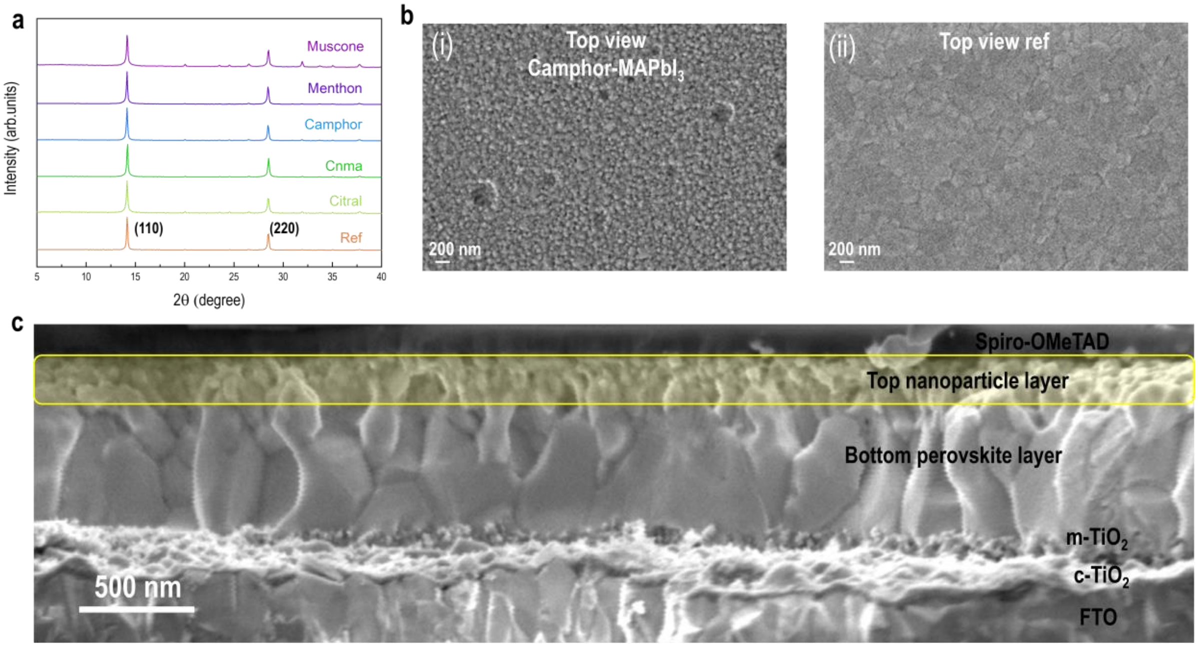

Supplementary Fig. 14. Thin film characterization for perovskite thin films with Tier 2 biomolecules. a X-ray diffraction (XRD) spectra of the pristine $MAPbI_3$ film and $MAPbI_3$ films doped with 3 mg mL^{-1} Tier 2 certain biomolecules. **b** Top-view SEM image of (i) Camphor-$MAPbI_3$ film showing the nanocrystal feature of the top layer, with comparison of (ii) a reference film (pristine-$MAPbI_3$). **c** Cross-section SEM image showing the stacking layer configuration of perovskite.

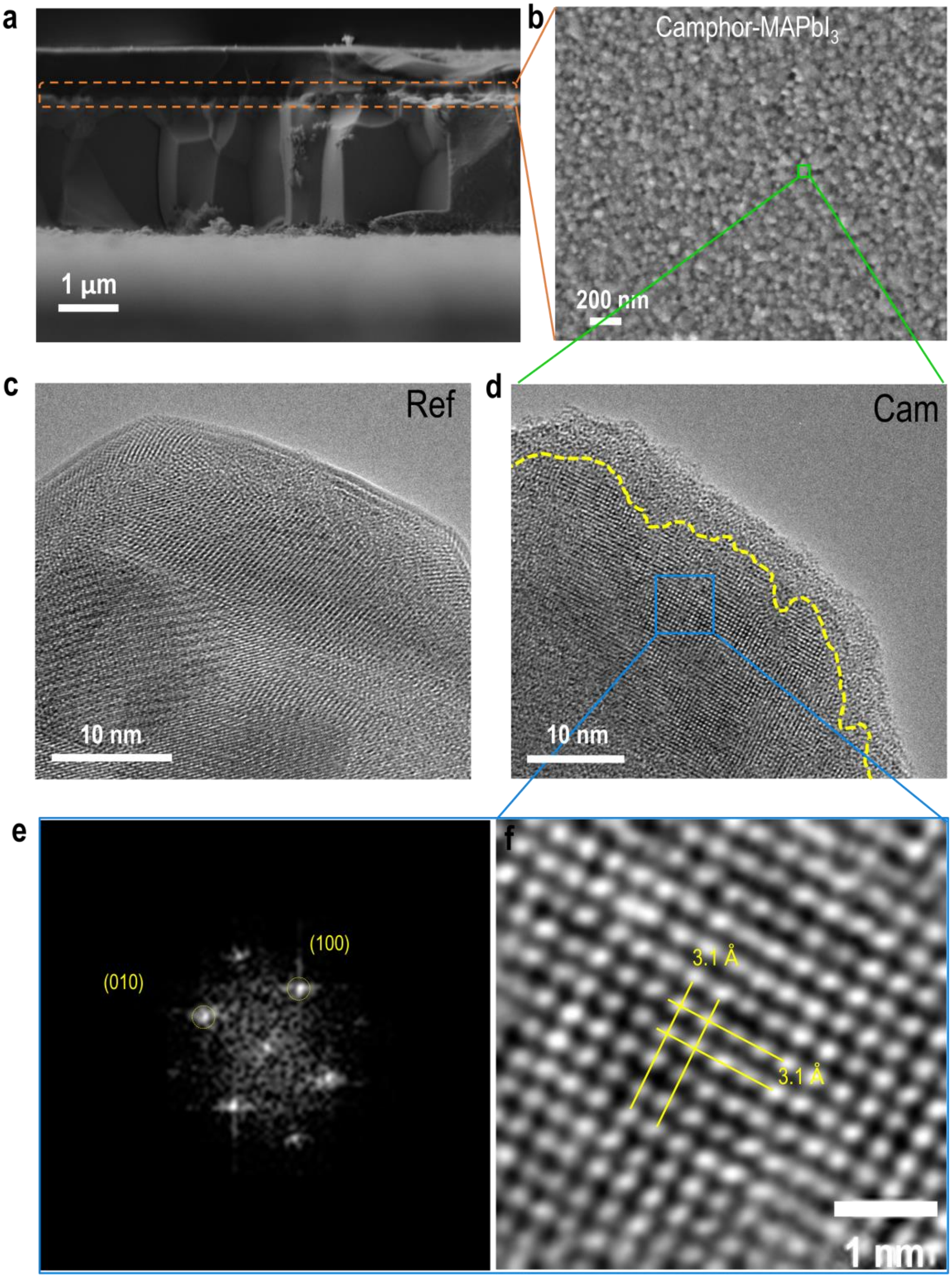

Supplementary Fig. 15. Thin film characterization for perovskite thin films with Tier 2 biomolecules. a Cross-section SEM image of perovskite solar cell with $MAPbI_3$ doped with 3 mg

mL^{-1} camphor. **b** Top-view SEM image of $MAPbI_3$ film doped with 3 mg mL^{-1} camphor. **c** Transmission electron microscopy (TEM) image of $MAPbI_3$ nanoparticle. **d** TEM image of $MAPbI_3$ nanoparticle wrapped with camphor biomolecules. **e** Fast Fourier transform (FFT) diffraction pattern of the high-resolution transmission electron microscopy (HRTEM) image in **f**, showing superlattice reflections at (100) and (010) planes. **f** HRTEM image of inner crystal lattice of the nanoparticle.

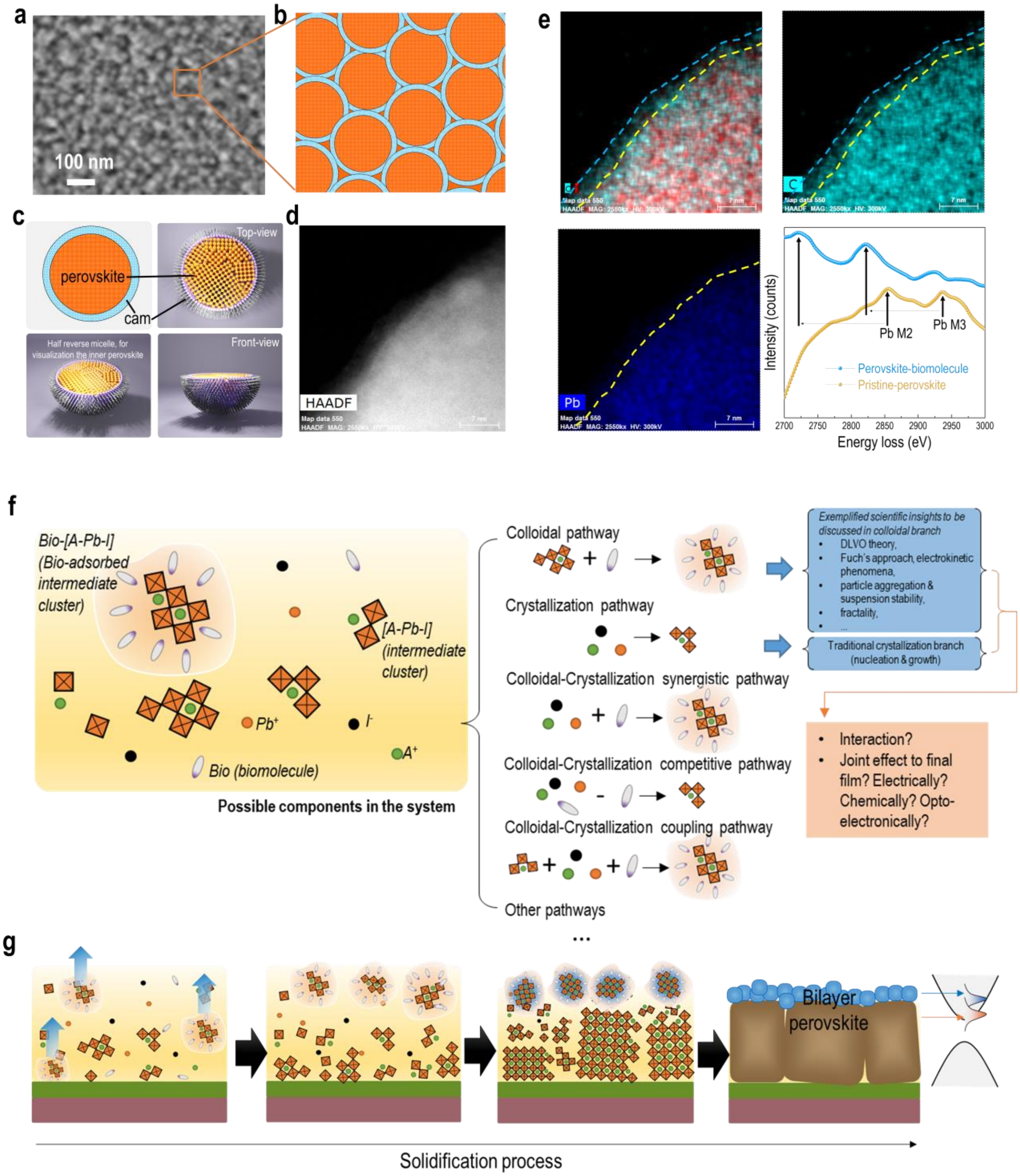

Supplementary Fig. 16. Formation mechanism of "reverse micelle" and "bilayer". a Top-view SEM image of $MAPbI_3$ film doped with 3 mg mL^{-1} camphor. **b** Schematic of nanocrystalline reverse micelle structure. **c** Illustration of perovskite-camphor reverse micelle structure (a half micelle is shown here to visualize the inner perovskite). **d** High-angle annular dark field (HAADF)

image of perovskite-camphor nanoparticle. **e** Elemental mapping of the $MAPbI_3$-camphor nanocrystal: where I and Pb are exclusively from perovskite while C is from both perovskite and camphor. The yellow dash line denotes the perovskite grain edge, while the blue dash line denotes the periphery of the particle. Electron energy loss spectroscopy (EELS) comparing perovskite-biomolecule vs. pristine-perovskite, to validify the chemical interaction from biomolecules to the Pb element in the perovskite. Briefly, the Pb M2 and M3 edges display a large shift after biomolecular-adsorption in the sample, which is in contrast to the case of pristine $MAPbI_3$. This means that there is a significant change in the coordination environment around Pb atom, which can be ascribed to the Pb-carbonyl group bonding between perovskite and the biomolecule. **f** Possible components in the co-colloidal-crystallization system containing perovskite precursors and biomolecules. Underlying fundamentals in this complex system in regarding the interaction between crystallization and colloidal dynamics, in-depth scientific insights that can be of interest in future research. **g** Hypothetical formation mechanism of the perovskite/perovskite bilayer structure where the top perovskite nanocrystals exhibit higher energy states whereby leading to a higher potential to the device.

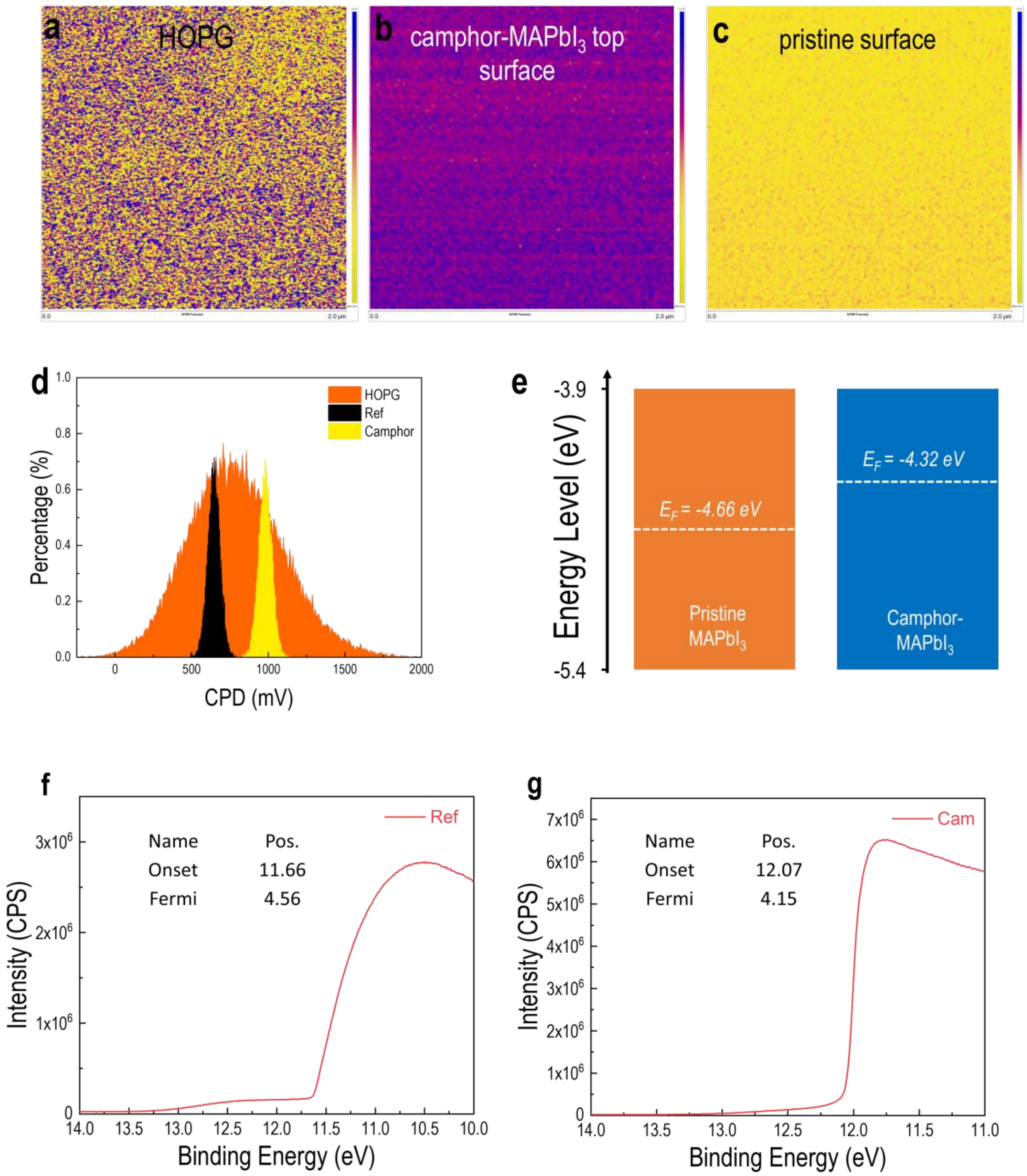

Supplementary Fig. 17. Energy level analysis for pristine perovskite and camphor-incorporated perovskite thin films. Contact potential difference (CPD) mapping of **a** highly oriented pyrolytic graphite (HOPG), **b** camphor-perovskite, **c** pristine $MAPbI_3$ surface. **d** CPD histogram spectra of HOPG, pristine $MAPbI_3$ and camphor-$MAPbI_3$ film. **e** Fermi level scheme of pristine $MAPbI_3$ and camphor-$MAPbI_3$ film. UPS spectra of **f** pristine perovskite film and **g** camphor-based perovskite film (under 5 V bias).

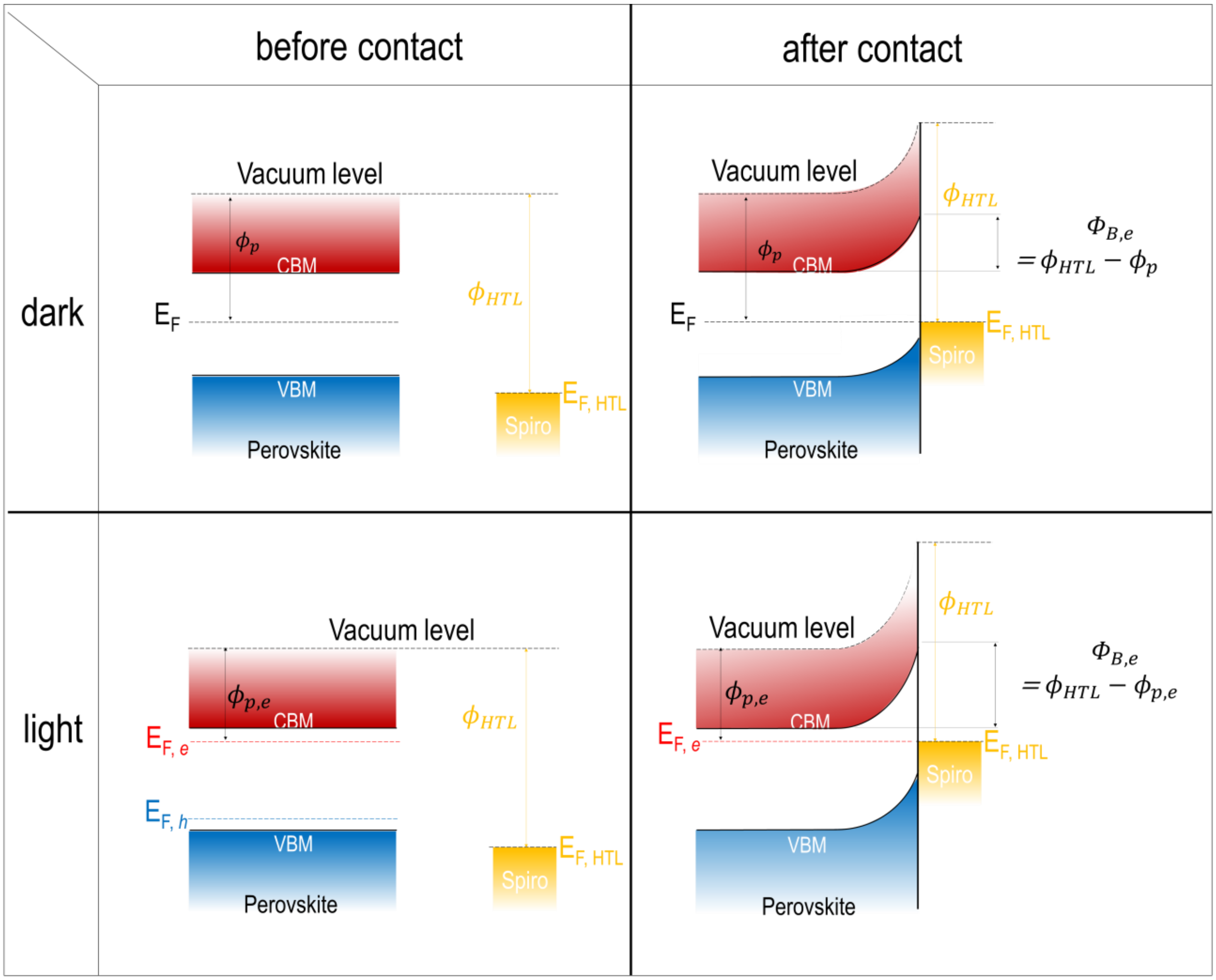

Supplementary Fig. 18. Energy level diagram of perovskite layer and spiro-OMeTAD layer before and after contact. In dark condition, after contacting, the conduction band (CBM) and valence band (VBM) of perovskite layer is bended and a Schottky barrier for electron is formed between the perovskite layer and spiro-OMeTAD layer (while for holes, there is an ohmic contract at this interface, so holes can be freely transfer across this heterojunction), an energy barrier $\Phi_{B,\,e} = \Phi_{HTL} - \Phi_p$ is established for blocking electron transfer from perovskite to spiro-OMeTAD HTL. In light condition, Fermi levels of electron and hole are separated due to the dynamic equilibrium condition. Similar to dark condition, holes can still have an ohmic contact and thus freely transfer across the interface. For electrons, there still exist the Schottky barrier, with a value of $\Phi_{B,\,e} = \Phi_{HTL} - \Phi_{p,\,e}$. Here the $\Phi_{B,\,e}$ is the quasi-Fermi level of electron under illumination. Hence, the similar barrier concept also exists under the light condition.

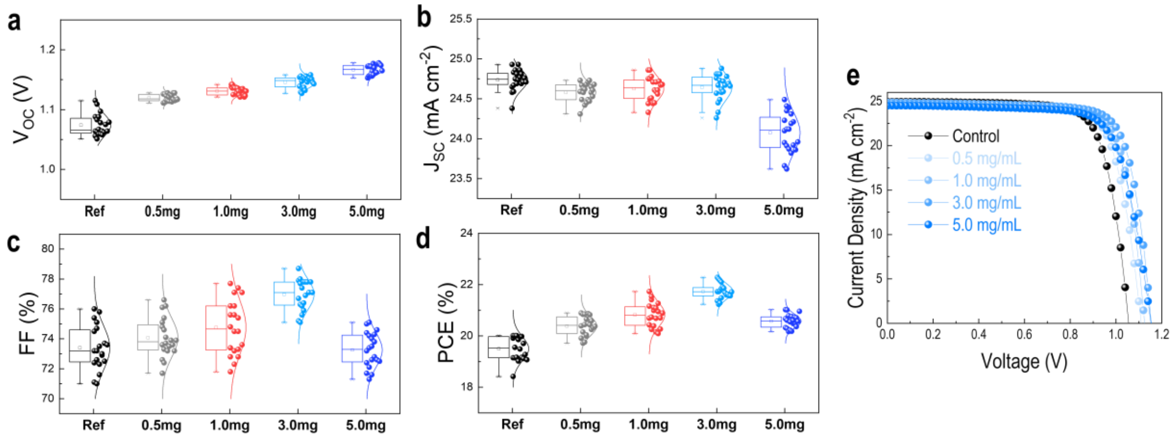

Supplementary Fig. 19. Device performance summary of perovskite solar cells with camphor at different doping concentrations. a-d Statistic data of photovoltaic parameters of perovskite solar cells with 0.5, 1.0, 3.0, 5.0 mg mL^{-1} camphor biomolecule. **e** J-V characteristics of perovskite solar cells with different concentrations of camphor biomolecule, 0.5, 1.0, 3.0, 5.0 mg mL^{-1}.

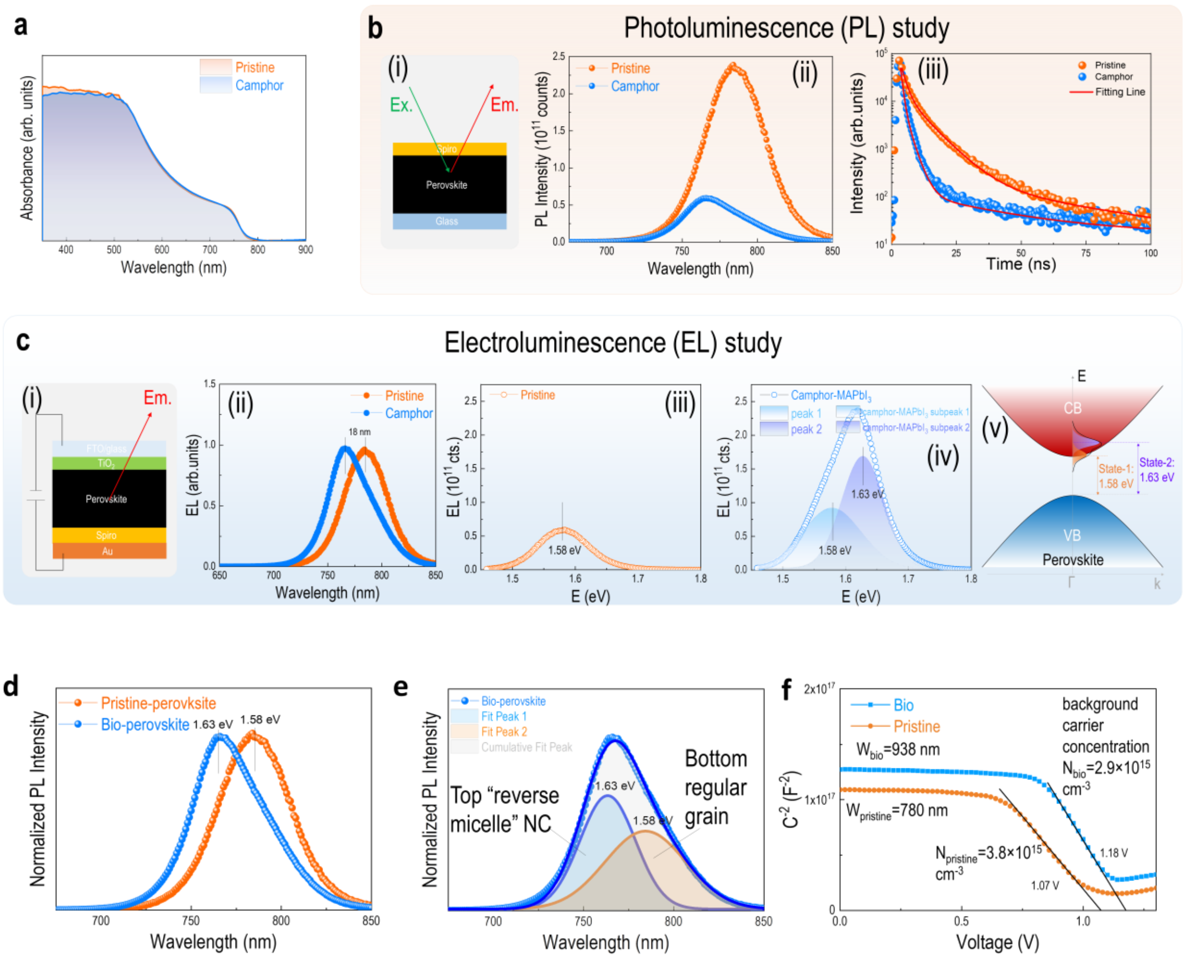

Supplementary Fig. 20. Thin film characterization for perovskite thin films with camphor biomolecules. a Absorption spectra of pristine $MAPbI_3$ film without or with camphor biomolecule. **b (i)** Schematic of photoluminescence (PL) measurement with a sample structure of glass/perovskite/spiro-OMeTAD. **(ii)** PL and **(iii)** time resolved photoluminescence (TRPL) spectra of pristine $MAPbI_3$ film without or with camphor biomolecule. **c (i)** Schematic of electroluminescence (EL) measurement with a sample structure of glass/TiO_2/perovskite/spiro-OMeTAD/Au. **(ii)** EL spectra of pristine $MAPbI_3$ film without or with camphor biomolecule. **(iii)** Peak position of EL spectra of pristine $MAPbI_3$ film at 1.58 eV. **(iv)** Peak position of EL spectra of $MAPbI_3$ film with camphor biomolecule, where two peaks at 1.58 eV and 1.63 eV are observed. **(v)** Schematic of energy level of $MAPbI_3$ film with camphor biomolecule, suggesting the presence of the higher energy states at 1.63 eV due to the presence of the top nanoparticle perovskite layer, while the lower state of 1.58 eV is from the bottom large crystal layer consistent to that from the pristine sample. **d, e** PL measurement and band gap estimation. **d** Normalized PL spectra of films of pristine-perovskite and bio-perovskites. **e** Two-peak fitting using Gaussian methods, where two

sub-spectra peaked at 1.63 and 1.58 eV are obtained, representing the top "reverse micelle" bio-perovskite nanocrystals layer and the bottom regular grain layer, respectively. **f** Mott-Schottky plots (C^{-2}-V) for devices using bio-perovskite and pristine perovskite, respectively. Built-in potential values, background carrier concentrations, and depletion width at 0 V are listed along with the curves.

As can be seen in Supplementary Figs. 20d and 20e, we measured the PL spectra of the bilayer perovskite sample. Compared to the pristine perovskite, the bio-perovskite shows two sub-peaks with peak position located at 765 and 784 nm, respectively. The 784 nm is consistent to the PL peak in the pristine-perovskite, which comes from the bottom regular perovskite crystalline grains. And the 765 nm can then be assigned to the top "reverse micelle" biomolecule -perovskite nanocrystal, i.e., a bandgap of 1.63 eV. Different from those in Si solar cell where p-type Si and n-type Si can form the Si-Si p-n junction, here between top and bottom perovskite there is no such p-n junction. Although we observe there is a slight Fermi level difference between them, while considering the whole device the junction at the interface between TiO_2/bottom perovskite and the top perovskite/Spiro can have more significant charge depletion at these interfaces. The depletion width obtained from typical method such as Mott-Schottky analysis is typically describing the depletion throughout the whole device, where the interface of electron transfer layer (ETL)/perovskite and perovskite/hole transfer layer (HTL) is predominantly manifested in the C-V plot.

Nevertheless, we also carried out the C-V measurement on our devices to see if the top bio-perovskite layer can have observable effects on the overall depletion width. The depletion width can be calculated from equation of[54]

$$W = (2\varepsilon_0\varepsilon_r V_{bi}/qN)^{1/2} \qquad \text{(S8)}$$

Where ε_0 is the vacuum permittivity, ε_r is the relative dielectric constant of the perovskites, V_{bi} is the build-in potentials of the device, q is the elementary charge and N is background carrier concentration, respectively. In order to determine the V_{bi} and N, we carried out the Mott-Schottky analysis using the capacitance-voltage (C-V) measurement. Supplementary Fig. 20f shows the C^{-2}-V plot, which can be derived to have a correlation of[55]

$$\frac{1}{C^2} = \frac{2(V_{bi} - V)}{A^2 q \varepsilon_0 \varepsilon_r N} \qquad \text{(S9)}$$

Where A is the active area, C is the capacitance, and V is the applied bias, respectively. From the slope in the linear region and the intersect of y-axis, both V_{bi} and N can be determined. The calculated results are presented in Supplementary Fig. 20f, where the bio-perovskite device exhibits a built-in potential of 1.18 V with a background carrier concentration of 2.9×10^{15} cm^{-3}, whereas the pristine perovskite device displays a smaller potential of 1.07 V with a concentration of 3.8×10^{15} cm^{-3}. The enlarged V_{bi} is consistent to the observations in the higher V_{OC} in the bio-incorporated device. While the reduced background carrier concentration can be ascribed to a de-

doping effect that we assume the biomolecules' incorporation reduced the self-doping related trap densities, which is consistent to the observation of reduced SRH recombination in our recombination studies. After getting both V_{bi} and N, we further calculate the depletion width at 0 V, which are also presented in the Supplementary Fig. 20f. The bio-perovskite device shows a larger depletion width of 938 nm compared to that of 780 nm in the pristine perovskite device. It should be noted that since we use the whole device for the C-V measurement, there are two heterojunctions at the interface of ETL/bottom perovskite layer and top perovskite layer/HTL (the bottom perovskite layer/top perovskite layer interface may contribute but not as significant as the interface with ETL and HTL, considering the larger energy level off-set at the interface with the charge transfer layers). Therefore, the larger depletion width from the bio-perovskite device is mostly related to a better top perovskite layer/HTL interface, as both devices have identical ETL/bottom perovskite layer interface.

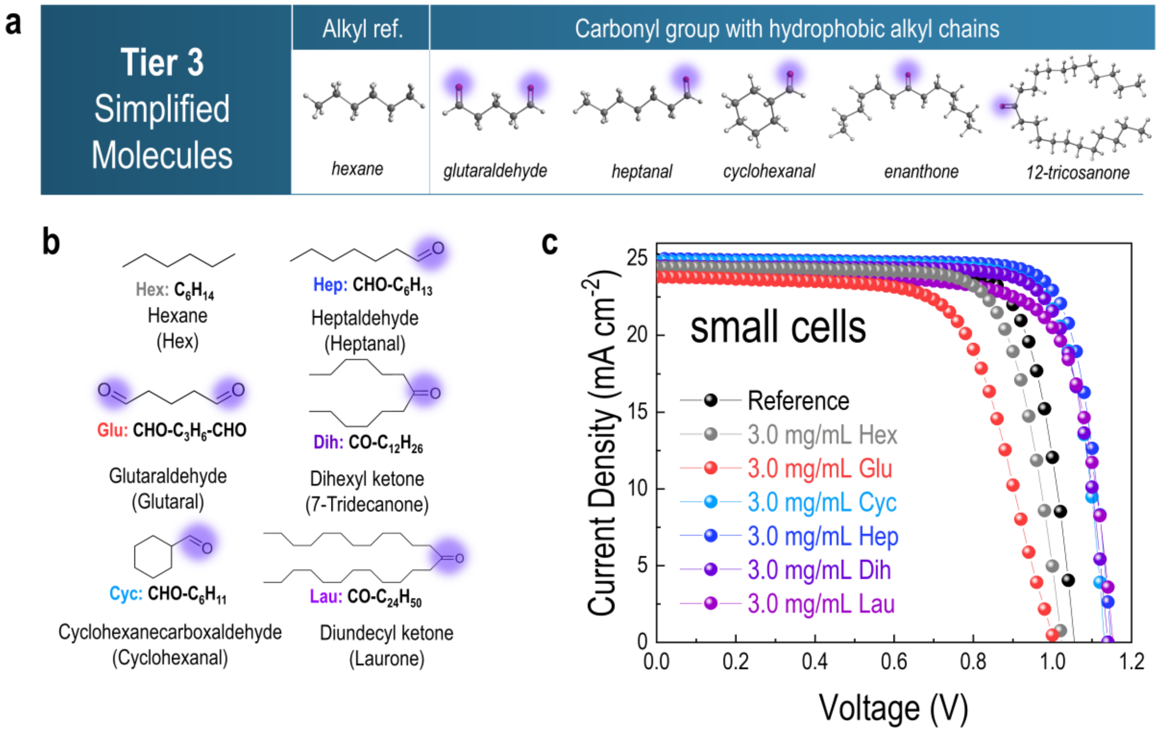

Supplementary Fig. 21. Schematic illustration of Tier 3 molecules and corresponding device performance with the addition of them. a, b Molecule structure of Tier 3 simplified molecules, including hexane (hexH, no carbonyl head), glutaraldehyde (glutaral, 2 carbonyl heads), cyclohexanecarboxaldehyde (cyclohexanal, 1 head and 1 cyclic tail), heptaldehyde (heptanal, 1 head and 1 linear tail), dihexyl ketone (7-tridecanone, 1 head and 2 linear tails), diundecyl ketone (laurone, 1 head and 2 long tails). **c** J-V characteristics of small-area perovskite solar cells with 3 mg mL^{-1} corresponding Tier 3 simplified molecule additives.

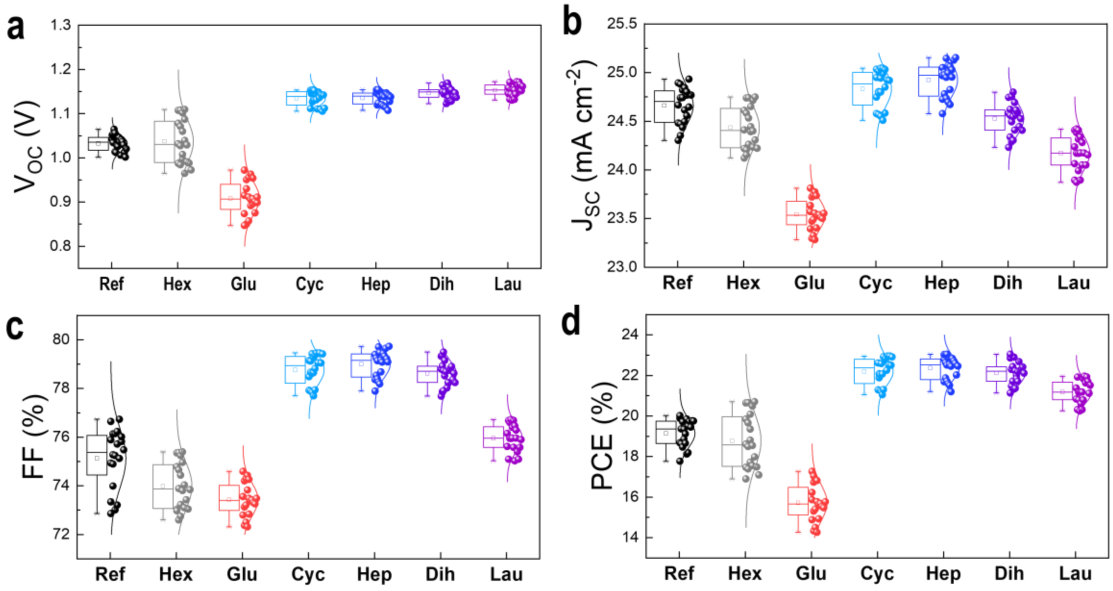

Supplementary Fig. 22. Device performance summary of perovskite solar cells with Tier 3 molecules. a-d Statistic data of photovoltaic parameters of perovskite solar cells with 3 mg mL^{-1} Tier 3 simplified molecule additives.

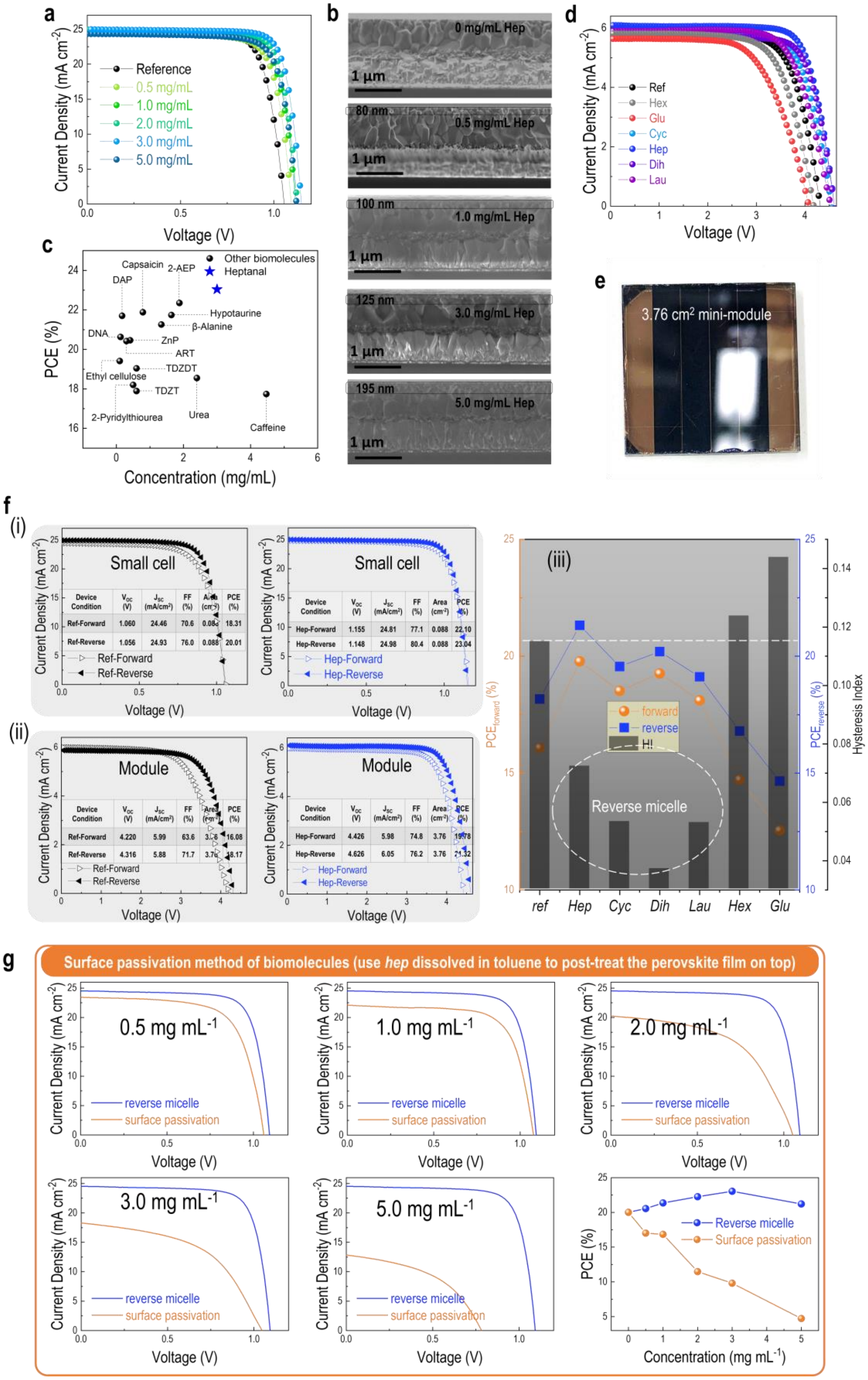

a
Current Density (mA cm-2)
Voltage (V)
Reference
0.5 mg/mL
1.0 mg/mL
2.0 mg/mL
3.0 mg/mL
5.0 mg/mL
b
0 mg/mL Hep
0.5 mg/mL Hep
1.0 mg/mL Hep
3.0 mg/mL Hep
5.0 mg/mL Hep
80 nm
100 nm
125 nm
195 nm
1 μm
c
PCE (%)
Concentration (mg/mL)
Other biomolecules
Heptanal
Capsaicin
2-AEP
DAP
Hypotaurine
β-Alanine
DNA
ZnP
ART
TDZDT
Ethyl cellulose
TDZT
2-Pyridylthiourea
Urea
Caffeine
d
Current Density (mA cm-2)
Voltage (V)
Ref
Hex
Glu
Cyc
Hep
Dih
Lau
e
3.76 cm2 mini-module
f
(i)
Small cell
Ref-Forward 1.060 24.46 70.6 0.088 18.31
Ref-Reverse 1.056 24.93 76.0 0.088 20.01
Hep-Forward 1.155 24.81 77.1 0.088 22.10
Hep-Reverse 1.148 24.98 80.4 0.088 23.04
(ii)
Module
Ref-Forward 4.220 5.99 63.6 3.76 16.08
Ref-Reverse 4.316 5.88 71.7 3.76 18.17
Hep-Forward 4.426 5.98 74.8 3.76 19.78
Hep-Reverse 4.626 6.05 76.2 3.76 21.32
(iii)
PCEforward (%)
PCEreverse (%)
Hysteresis Index
forward
reverse
HI
Reverse micelle
ref
Hep
Cyc
Dih
Lau
Hex
Glu
g
Surface passivation method of biomolecules (use hep dissolved in toluene to post-treat the perovskite film on top)
0.5 mg mL-1
1.0 mg mL-1
2.0 mg mL-1
3.0 mg mL-1
5.0 mg mL-1
reverse micelle
surface passivation
Current Density (mA cm-2)
Voltage (V)
PCE (%)
Concentration (mg mL-1)
Reverse micelle
Surface passivation

Supplementary Fig. 23. Device performance characterization of perovskite solar cells with Tier 3 molecules. a J-V characteristics of small-area perovskite solar cells using heptanal additive of different concentrations from 0.5 to 1.0, 2.0, 3.0, and 5.0 mg mL^{-1}. **b** SEM images of pristine $MAPbI_3$ and heptanal-$MAPbI_3$ perovskite films of different additive concentrations. **c** PCE survey plot of perovskite solar cells with different biomolecule additives with different doping concentrations, reference detailed in Supplementary Note 4. **d** J-V characteristics of perovskite solar minimodules with corresponding Tier 3 simplified molecule additives. **e** Photo of a 3×3 cm^2 perovskite solar minimodules from the backside (device active area 3.76 cm^2). **f** Hysteresis investigation. We checked both the small area and module devices: the Hep-devices show smaller hysteresis in both (i) small area cell devices and (ii) mini-module devices. (iii) Module level comparison on PCEs scanned at different directions and hysteresis index, calculated from equation of $HI = \frac{PCE_{reverse\ scan} - PCE_{forward\ scan}}{PCE_{reverse\ scan}}$ (Supplementary Equation 10),[56] where $PCE_{reverse\ scan}$ is the module PCEs obtained from reverse scan and $PCE_{forward\ scan}$ is the same module PCEs obtained from forward scan. All the "reverse micelle" related biomolecules (Hep, Cyc, Dih, Lau) exhibit lower HI compared to reference, whereas the nonpolar Hex and bipolar Glu show higher HI. The reduced HI in "reverse micelle" device can be ascribed to the terminal atomic anchoring effect by the ligand which minimizes the ionic contribution in the perovskite. **g** J-V curves of perovskite solar cells using different heptanal concentrations and a concentration dependent PCE plot comparing the result from two processing of "reverse micelle" method (dissolve the biomolecule in the solution, followed by a self-assembly to form reverse micelle and bilayer film), and "surface passivation" method (use biomolecule dissolved in toluene to post-treat the perovskite film on top).

a

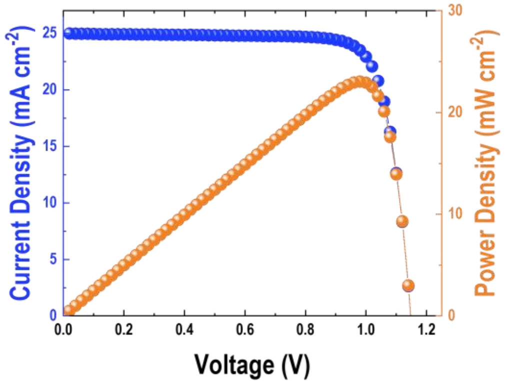

Device Condition	V_{OC} (V)	J_{SC} (mA/cm^2)	FF (%)	Area (cm^2)	PCE (%)	Power (mW)
3.0 mg/mL Hep	1.148	24.98	80.4	0.088	23.04	20.28

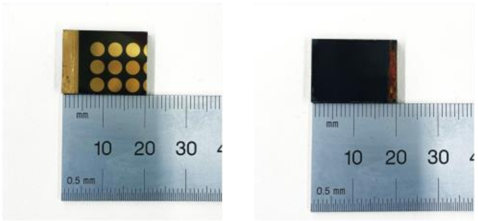

0.088 cm^2 mini-module (single cells)

b

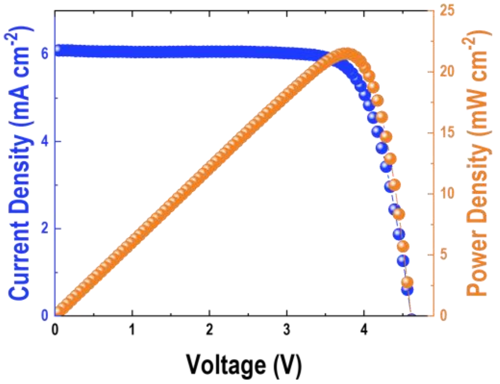

Device Condition	V_{OC} (V)	J_{SC} (mA/cm^2)	FF (%)	Area (cm^2)	PCE (%)	Power (mW)
3.0 mg/mL Hep	4.626	6.05	76.2	3.76	21.32	80.19

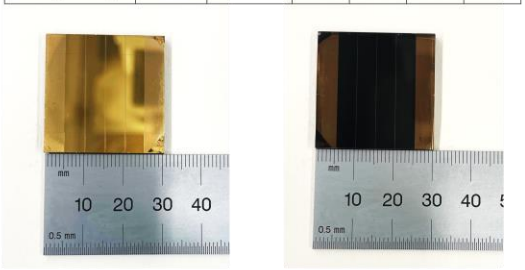

3.76 cm^2 mini-module (4-sub cells)

c

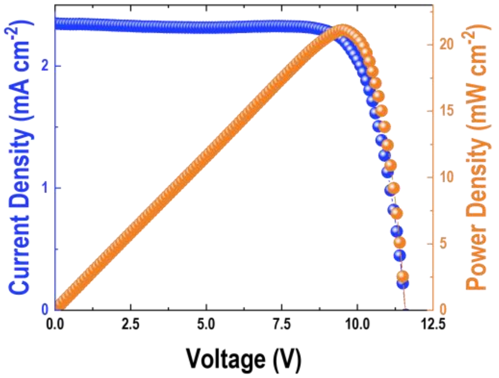

Device Condition	V_{OC} (V)	J_{SC} (mA/cm^2)	FF (%)	Area (cm^2)	PCE (%)	Power (mW)
3.0 mg/mL Hep	11.594	2.34	75.6	23.5	20.51	481.99

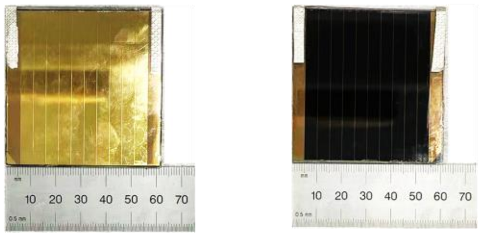

23.5 cm^2 mini-module (10-sub cells)

d

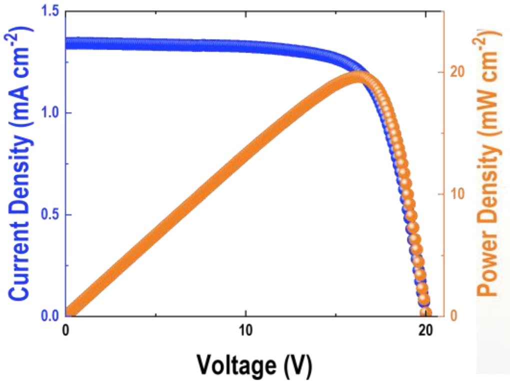

Device Condition	V_{OC} (V)	J_{SC} (mA/cm^2)	FF (%)	Area (cm^2)	PCE (%)	Power (mW)
3.0 mg/mL Hep	20.230	1.36	73.2	70.0	20.14	1409.76

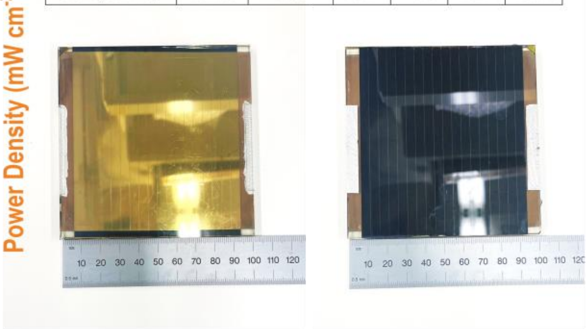

70 cm^2 mini-module (18-sub cells)

Supplementary Fig. 24. Module level device performance demonstration. J-V characteristics, photovoltaic parameters, photo images form device front side and backside of **a** small-size cells, **b** 3×3 cm^2 modules, **c** 6×6 cm^2 modules, and **d** 10×10 cm^2 modules.

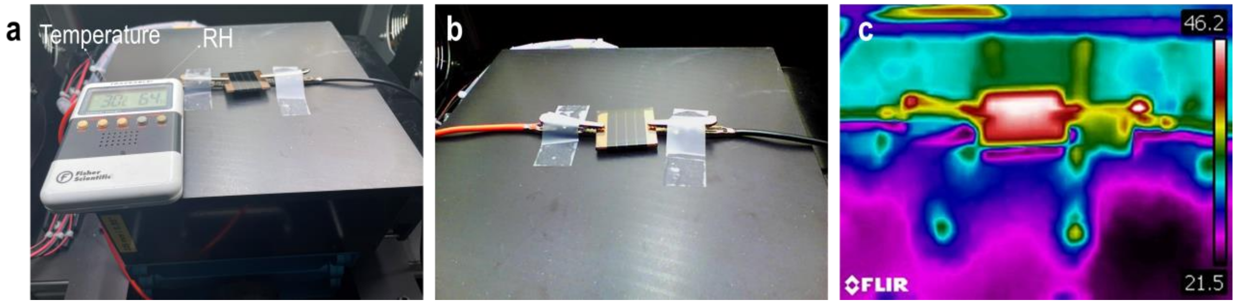

Supplementary Fig. 25. Testing environment for MPPT. a Exemplified photo image of MPPT measurement in ambient environment (30 ℃, 64% RH). **b** Photo image of 3×3 cm^2 module device during the MPPT. **c** Thermal image of the 3×3 cm^2 module device during the MPPT.

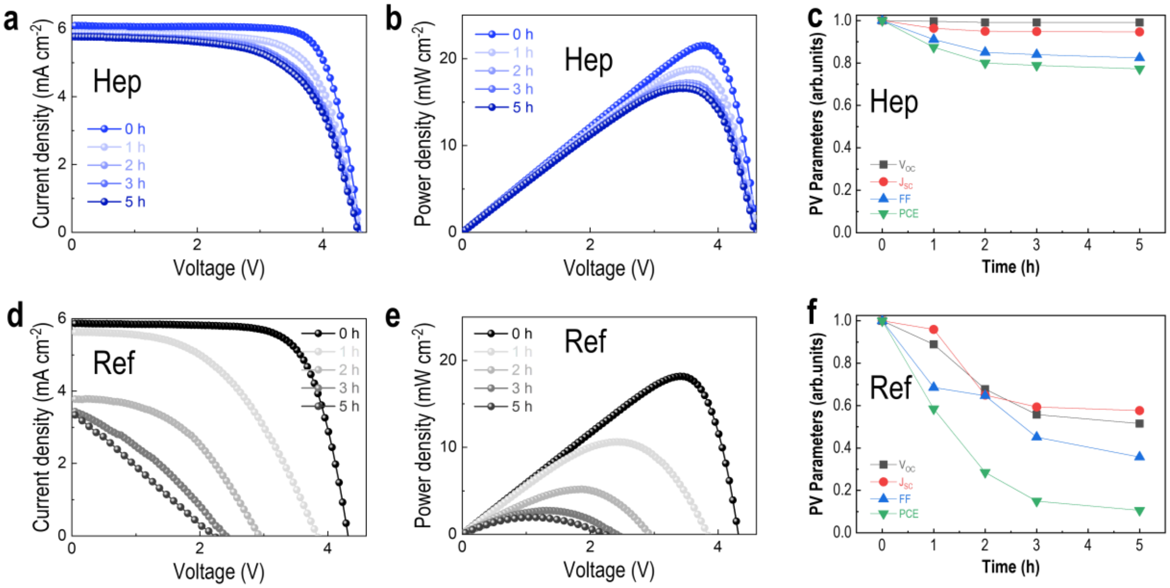

Supplementary Fig. 26. Light stability test for perovskite solar cells with or without heptanal molecule as additive. **a** J-V characteristics, **b** P-V characteristics, and **c** photovoltaic parameters of 3×3 cm^2 perovskite mini-module with heptanal additive after illumination of 0, 1, 2, 3, 5 hours. **d** J-V characteristics, **e** P-V characteristics, and **f** photovoltaic parameters of 3×3 cm^2 reference perovskite mini-module after illumination of 0, 1, 2, 3, 5 hours.

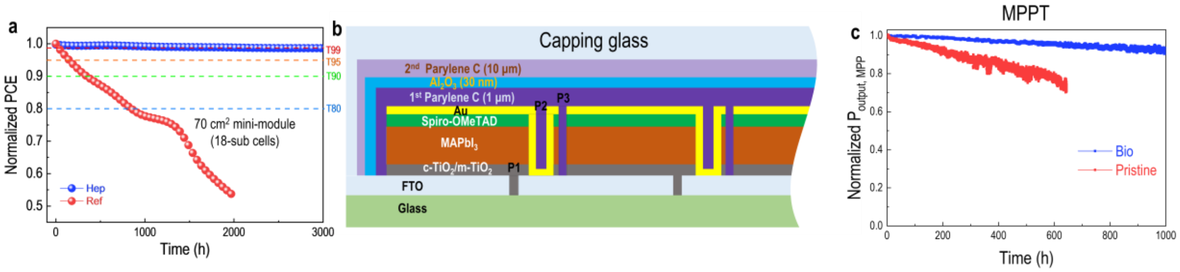

Supplementary Fig. 27. Stability test for perovskite solar modules. a Shelf-stability measurement of encapsulated 10×10 cm^2 (FTO substrate size, with an active area of 70 cm^2) perovskite sub-modules with or without heptanal additive under ambient environment. **b** Scheme of device structure with encapsulation details. **c** MPPT of encapsulated devices in air (20-30% RH, room temperature, cooling stage equipped, encapsulation process is articulated in the method section). We used 1-Sun equivalent white LED illumination as light source. Initial MPP power output is consistent to the calculated power from I-V measurement, suggesting well matched spectrum of the light source.

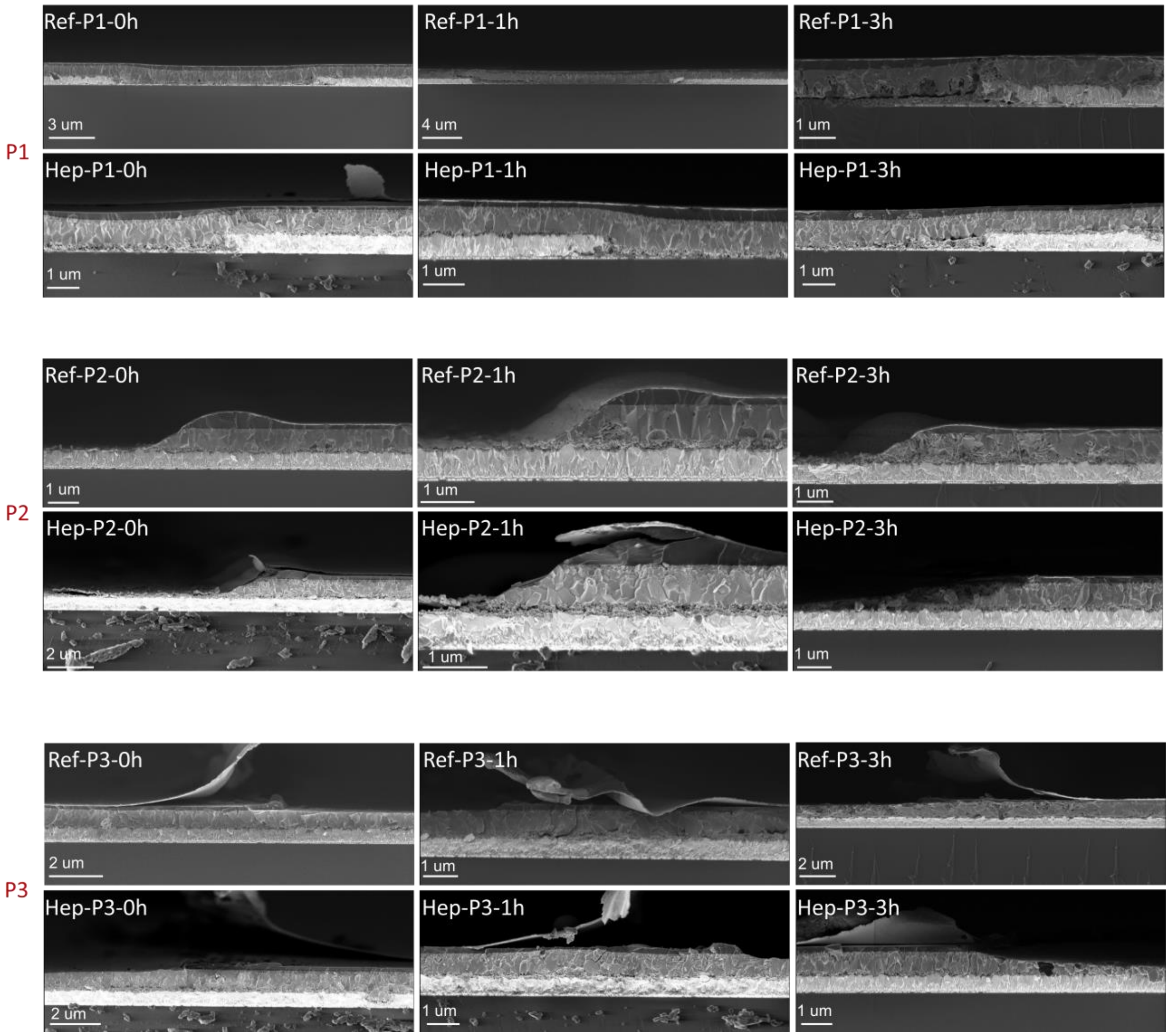

Supplementary Fig. 28. SEM images of perovskite modules illuminated continuously after 0, 1, 3h and measured at different locations of P1, P2, P3 channels. Comparison is between a pristine module and a heptanal incorporated module. Cracks and pores are found in the pristine device after aging.

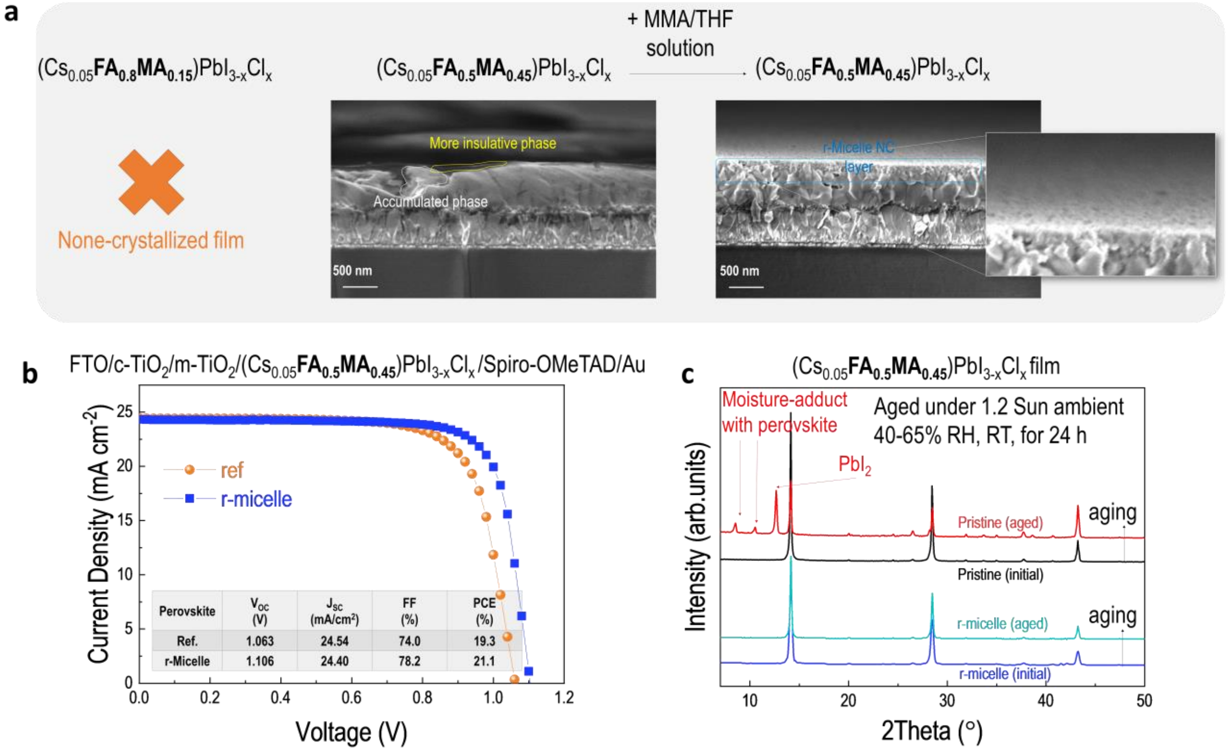

Supplementary Fig. 29. Film and device features of Cs-doped MA-FA system with bio-additive. a Solution compositional engineering to realize "reverse micelle" structure in Cs-doped MA-FA system. Cross-sectional SEM images verifying the concept. Preliminary results on r-micelle effect on **b** device efficiency (J-V curve of Cs-doped MA-FA system with and without r-micelle structures) and **c** stability (XRD spectra comparing fresh and aged samples with or without biomolecular treatment).

Supplementary Tables 1-7

Supplementary Table 1. Characteristics of five types of common biomolecules in biosystems.

Group name	Chemical formula	Structural formula	Ball-and-stick model	Properties in bio	Common functions	Commonly found in
hydroxyl	—OH	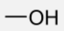	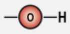	• polar<br>• hydrophilic	tends to make things more soluble in water	• hugely abundant in sugars and alcohols
carboxyl	—COOH	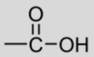	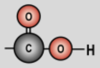	• acidic<br>• charged(-)	multifaceted, biological acid	• amino acids (proteins)<br>• fatty acids<br>• acetic acids<br>• other acids
amino	$—NH_2$	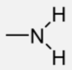	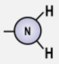	• basic<br>• charged(+)	biological base, maintains 3d structure of large molecules, defines base pairs in nucleic acids	• amino acids (proteins)<br>• neurotransmitters<br>• bases of nucleic acids
phosphate	$—PO_4$	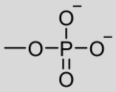	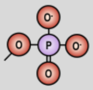	• acidic<br>• charged(-)<br>• hydrophilic	regulation, energy, structure, DNA	• phospholipids<br>• DNA backbone<br>• NTP, ATP<br>• protein regulation
carbonyl	—COH<br>—CO—	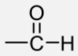<br>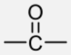	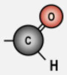<br>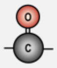	• polar	the site of enzymatic bond breaking/making	• aldehydes & ketones<br>• lipid<br>• sugar

Supplementary Table 2. Characteristic comparisons of Tier 1 (general) biomolecules (under the best concentration) in different aspects (cost, device performance, solubility, hydrophobicity), photovoltaic parameters statistics can be seen from Supplementary Fig. 11.

Material (mg mL^{-1})	Cost ($ g^{-1})	T80 (h)	PCE (%)	J_{SC} (mA cm^{-2})	V_{OC} (V)	FF (%)	Water solubility	Hydrophobicity
Reference	31	5.53	18.22±0.60	23.38±0.29	1.054±0.020	73.6±2.2	Intermediate soluble	2.5
Niacin-0.1	0.09	3.07	16.96±0.99	22.32±0.45	1.030±0.016	73.8±3.7	Very soluble	0.5
β-Estradiol-1	33.8	3.56	20.65±0.56	24.31±0.30	1.101±0.007	77.2±2.2	25 mg mL^{-1}, clear to slightly hazy	3.5
NAM-0.5	0.11	17.01	18.52±0.44	23.17±0.09	1.066±0.020	75.0±1.7	1 g is soluble in 1 mL water 5/5	1.0
DNA-0.05	83.3	23.36	19.28±0.79	24.07±0.33	1.062±0.017	75.4±2.9	DNA is water soluble 3/5	2.0
ART-0.1	840	60	21.04±0.69	24.20±0.76	1.129±0.008	77.0±1.5	Not soluble 1/5	5.0

Supplementary Table 3. Photovoltaic parameters of devices (champion PCE) with Tier 2 (certain) biomolecules (statistics can be seen from Supplementary Figs. 12b-e).

Device Condition	V_{OC} (V)	J_{SC} (mA cm^{-2})	FF (%)	PCE (%)
Reference	1.056	24.93	76.0	20.01
3.0 mg mL^{-1} Citral	1.138	24.37	73.9	20.51
3.0 mg mL^{-1} Cnma	1.182	24.61	75.6	22.00
3.0 mg mL^{-1} Camphor	1.154	24.76	77.9	22.28
3.0 mg mL^{-1} Menthon	1.190	25.10	71.0	21.21
3.0 mg mL^{-1} Muscone	1.176	23.96	75.8	21.37

Supplementary Table 4. Photovoltaic parameters of devices (champion PCE) with camphor biomolecule of different concentrations (statistics can be seen from Supplementary Figs. 19a-d).

Device Condition	V_{OC} (V)	J_{SC} (mA cm^{-2})	FF (%)	PCE (%)
Reference	1.056	24.93	76.0	20.01
0.5 mg mL^{-1} Camphor	1.111	24.73	76.1	20.89
1.0 mg mL^{-1} Camphor	1.125	24.86	77.7	21.73
3.0 mg mL^{-1} Camphor	1.154	24.76	77.9	22.28
5.0 mg mL^{-1} Camphor	1.153	24.49	74.4	21.02

Supplementary Table 5. Photovoltaic parameters of devices (champion PCE) with Tier 3 (simplified) molecules (statistics can be seen from Supplementary Fig. 22).

Device Condition	V_{OC} (V)	J_{SC} (mA cm^{-2})	FF (%)	PCE (%)
Reference	1.056	24.93	76.0	20.01
3.0 mg mL^{-1} Hep	1.148	24.98	80.4	23.04
3.0 mg mL^{-1} Cyc	1.131	24.81	78.9	22.13
3.0 mg mL^{-1} Dih	1.140	24.51	78.8	22.03
3.0 mg mL^{-1} Lau	1.153	24.05	75.0	20.81
3.0 mg mL^{-1} Hex	1.023	24.44	74.9	18.74
3.0 mg mL^{-1} Glu	1.007	23.81	66.4	15.93

Supplementary Table 6. Photovoltaic parameters of devices (champion PCE) with heptanal of different concentrations.

Device Condition	V_{OC} (V)	J_{SC} (mA cm^{-2})	FF (%)	PCE (%)
Reference	1.056	24.93	76.0	20.01
0.5 mg mL^{-1} Hep	1.094	24.53	76.6	20.55
1.0 mg mL^{-1} Hep	1.117	24.48	78.1	21.36
2.0 mg mL^{-1} Hep	1.125	24.98	79.2	22.26
3.0 mg mL^{-1} Hep	1.148	24.98	80.4	23.04
5.0 mg mL^{-1} Hep	1.121	24.33	77.7	21.21

Supplementary Table 7. Photovoltaic parameters of perovskite mini-modules (champion PCE) with Tier 3 (simplified) molecules.

Device Condition	V_{OC} (V)	J_{SC} (mA cm^{-2})	FF (%)	PCE (%)
Reference	4.316	5.88	71.7	18.17
3.0 mg mL^{-1} Hep	4.626	6.05	76.2	21.32
3.0 mg mL^{-1} Cyc	4.555	5.79	74.1	19.56
3.0 mg mL^{-1} Dih	4.608	5.73	76.5	20.20
3.0 mg mL^{-1} Lau	4.531	5.90	71.5	19.12
3.0 mg mL^{-1} Hex	4.179	5.81	69.1	16.80
3.0 mg mL^{-1} Glu	4.082	5.65	63.7	14.66

Supplementary References

1. Melitz, W. et al. Scanning tunneling spectroscopy and Kelvin probe force microscopy investigation of Fermi energy level pinning mechanism on InAs and InGaAs clean surfaces. *J. Appl. Phys.* **108**, 023711 (2010).
2. Yang, D. et al. High efficiency planar-type perovskite solar cells with negligible hysteresis using EDTA-complexed SnO_2. *Nat. Commun.* **9**, 1-11 (2018).
3. Wang, K. et al. Nonionic $Sc_3N@C_{80}$ dopant for efficient and stable halide perovskite photovoltaics. *ACS Energy Lett.* **4**, 1852-1861 (2019).
4. Kao, K. C. *Dielectric Phenomena In Solids*. (Elsevier, Waltham, 2004).
5. Wang, Y. et al. Pushing the Envelope: Achieving an Open-Circuit Voltage of 1.18 V for Unalloyed $MAPbI_3$ Perovskite Solar Cells of a Planar Architecture. *Adv. Funct. Mater.* **28**, 1801237 (2018).
6. Wetzelaer, G. J. A. et al. Trap-assisted non-radiative recombination in organic–inorganic perovskite solar cells. *Adv. Mater.* **27**, 1837-1841 (2015).
7. Hegedus, S. S. & Shafarman, W. N. Thin-film solar cells: device measurements and analysis. *Prog. Photovolt. Res. Appl.* **12**, 155-176 (2004).
8. Alsalloum, A. Y. et al. Low-temperature crystallization enables 21.9% efficient single-crystal $MAPbI_3$ inverted perovskite solar cells. *ACS Energy Lett.* **5**, 657-662 (2020).
9. Chen, Y. et al. Dual passivation of perovskite and SnO_2 for high-efficiency $MAPbI_3$ perovskite solar cells. *Adv. Sci.* **8**, 2001466 (2021).
10. Chen, Z. et al. Single-crystal $MAPbI_3$ perovskite solar cells exceeding 21% power conversion efficiency. *ACS Energy Lett.* **4**, 1258-1259 (2019).
11. Choi, Y. et al. Toward All-Vacuum-Processable Perovskite Solar Cells with High Efficiency, Stability, and Scalability Enabled by Fluorinated Spiro-OMeTAD through Thermal Evaporation. *Sol. RRL* **5**, 2100415 (2021).
12. Gao, X. X. et al. High-Mobility Hydrophobic Conjugated Polymer as Effective Interlayer for Air-Stable Efficient Perovskite Solar Cells. *Sol. RRL* **3**, 1800232 (2019).
13. He, Q. et al. Highly Efficient and Stable Perovskite Solar Cells Enabled by Low-Cost Industrial Organic Pigment Coating. *Angew. Chem. Int. Edit.* **60**, 2485-2492 (2021).
14. Jeon, N. J. et al. o-Methoxy substituents in spiro-OMeTAD for efficient inorganic–organic hybrid perovskite solar cells. *J. Am. Chem. Soc.* **136**, 7837-7840 (2014).
15. Li, M. et al. Perovskite grains embraced in a soft fullerene network make highly efficient flexible solar cells with superior mechanical stability. *Adv. Mater.* **31**, 1901519 (2019).
16. Lin, H. S. et al. Triarylamine/Bithiophene Copolymer with Enhanced Quinoidal Character as Hole-Transporting Material for Perovskite Solar Cells. *Angew. Chem. Int. Edit.*, e202203949 (2022).
17. Molina-Ontoria, A. et al. Benzotrithiophene-Based Hole-Transporting Materials for 18.2% Perovskite Solar Cells. *Angew. Chem.* **128**, 6378-6382 (2016).
18. Ono, L. K. et al. Pinhole-free hole transport layers significantly improve the stability of $MAPbI_3$-based perovskite solar cells under operating conditions. *J. Mater. Chem. A* **3**, 15451-15456 (2015).
19. Turkevych, I. et al. Strategic advantages of reactive polyiodide melts for scalable perovskite photovoltaics. *Nat. Nanotech.* **14**, 57-63 (2019).

20. Wang, F. et al. Iodine-assisted antisolvent engineering for stable perovskite solar cells with efficiency> 21.3%. *Nano Energy* **67**, 104224 (2020).
21. Wu, Y. et al. Thermally stable $MAPbI_3$ perovskite solar cells with efficiency of 19.19% and area over 1 cm^2 achieved by additive engineering. *Adv. Mater.* **29**, 1701073 (2017).
22. Xiong, S. et al. Direct observation on p-to n-type transformation of perovskite surface region during defect passivation driving high photovoltaic efficiency. *Joule* **5**, 467-480 (2021).
23. Ye, X. et al. Er@C_{82} as a Bifunctional Additive to the Spiro-OMeTAD Hole Transport Layer for Improving Performance and Stability of Perovskite Solar Cells. *Sol. RRL* **5**, 2100463 (2021).
24. Zhu, G. et al. Unveiling the Critical Role of Oxidants and Additives in Doped Spiro-OMeTAD toward Stable and Efficient Perovskite Solar Cells. *ACS Appl. Energy Mater.* **5**, 3595-3604 (2022).
25. Castriotta, L. A. et al. Reducing Losses in Perovskite Large Area Solar Technology: Laser Design Optimization for Highly Efficient Modules and Minipanels. *Adv. Energy Mater.* **12**, 2103420 (2022).
26. Chen, S. et al. Stabilizing perovskite-substrate interfaces for high-performance perovskite modules. *Science* **373**, 902-907 (2021).
27. Di Giacomo, F. et al. Up-scalable sheet-to-sheet production of high efficiency perovskite module and solar cells on 6-in. substrate using slot die coating. *Sol. Energ. Mat. Sol. C.* **181**, 53-59 (2018).
28. Ding, Y. et al. Single-crystalline TiO_2 nanoparticles for stable and efficient perovskite modules. *Nat. Nanotech.*, 1-8 (2022).
29. Du, M. et al. High-pressure nitrogen-extraction and effective passivation to attain highest large-area perovskite solar module efficiency. *Adv. Mater.* **32**, 2004979 (2020).
30. Grancini, G. et al. One-Year stable perovskite solar cells by 2D/3D interface engineering. *Nat. Commun.* **8**, 1-8 (2017).
31. Han, G. S. et al. Spin-coating process for 10 cm× 10 cm perovskite solar modules enabled by self-assembly of SnO_2 nanocolloids. *ACS Energy Lett.* **4**, 1845-1851 (2019).
32. Jeong, M. et al. Large-area perovskite solar cells employing spiro-Naph hole transport material. *Nat. Photonics* **16**, 119-125 (2022).
33. Kim, M. et al. Conformal quantum dot–SnO_2 layers as electron transporters for efficient perovskite solar cells. *Science* **375**, 302-306 (2022).
34. Li, Z. et al. Organometallic-functionalized interfaces for highly efficient inverted perovskite solar cells. *Science* **376**, 416-420 (2022).
35. Lin, X. et al. In situ growth of graphene on both sides of a Cu–Ni alloy electrode for perovskite solar cells with improved stability. *Nat. Energy*, 1-8 (2022).
36. Liu, Z. et al. A holistic approach to interface stabilization for efficient perovskite solar modules with over 2,000-hour operational stability. *Nat. Energy* **5**, 596-604 (2020).
37. Rana, P. J. S. et al. Alkali Additives Enable Efficient Large Area (> 55 cm^2) Slot-Die Coated Perovskite Solar Modules. *Adv. Funct. Mater.*, 2113026 (2022).
38. Xiao, K. et al. Scalable processing for realizing 21.7%-efficient all-perovskite tandem solar modules. *Science* **376**, 762-767 (2022).
39. Zhou, W. et al. An Improbable Amino-Functionalized Fullerene Spacer Enables 2D/3D Hybrid Perovskite with Enhanced Electron Transport in Solar Cells. *Adv. Funct. Mater.*, 2201374 (2022).

40. Zhu, J. et al. Defect Healing in FAPb($I_{1-x}Br_x$)$_3$ Perovskites: Multifunctional Fluorinated Sulfonate Surfactant Anchoring Enables> 21% Modules with Improved Operation Stability. *Adv. Energy Mater.* **12**, 2200632 (2022).
41. Zhu, X. et al. Ionic-Liquid-Perovskite Capping Layer for Stable 24.33%-Efficient Solar Cell. *Adv. Energy Mater.* **12**, 2103491 (2022).
42. Hou, Y. et al. Enhanced performance and stability in DNA-perovskite heterostructure-based solar cells. *ACS Energy Lett.* **4**, 2646-2655 (2019).
43. Hou, Y. et al. Artemisinin (ART)-Induced "perovskite/perovskite" bilayer structured photovoltaics. *Nano Energy* **78**, 105133 (2020).
44. Lee, J. W. et al. A bifunctional lewis base additive for microscopic homogeneity in perovskite solar cells. *Chem* **3**, 290-302 (2017).
45. Li, C. et al. Monoammonium porphyrin for blade-coating stable large-area perovskite solar cells with> 18% efficiency. *J. Am. Chem. Soc.* **141**, 6345-6351 (2019).
46. Ling, X. et al. Guanidinium-assisted surface matrix engineering for highly efficient perovskite quantum dot photovoltaics. *Adv. Mater.* **32**, 2001906 (2020).
47. Sun, M. et al. Tuning the crystal growth of perovskite thin-films by adding the 2-pyridylthiourea additive for highly efficient and stable solar cells prepared in ambient air. *J. Mater. Chem. A* **5**, 13448-13456 (2017).
48. Wang, R. et al. Caffeine improves the performance and thermal stability of perovskite solar cells. *Joule* **3**, 1464-1477 (2019).
49. Yang, J. et al. Extremely low-cost and green cellulose passivating perovskites for stable and high-performance solar cells. *ACS Appl. Mater. Interfaces* **11**, 13491-13498 (2019).
50. Zhao, Y. et al. Molecular interaction regulates the performance and longevity of defect passivation for metal halide perovskite solar cells. *J. Am. Chem. Soc.* **142**, 20071-20079 (2020).
51. Zhu, H., Zhang, F., Xiao, Y., Wang, S. & Li, X. Suppressing defects through thiadiazole derivatives that modulate $CH_3NH_3PbI_3$ crystal growth for highly stable perovskite solar cells under dark conditions. *J. Mater. Chem. A* **6**, 4971-4980 (2018).
52. Moroi, Y. Micelles: theoretical and applied aspects. (Springer Science & Business Media, 1992).
53. Chandler, D. Interfaces and the driving force of hydrophobic assembly. *Nature* **437**, 640-647 (2005).
54. Fang, L. et al. Experimental and theoretical evidence of enhanced ferromagnetism in sonochemical synthesized $BiFeO_3$ nanoparticles. *Appl. Phys. Lett.* **97**, 242501 (2010).
55. Brown, P. R. et al. Improved current extraction from ZnO/PbS quantum dot heterojunction photovoltaics using a MoO_3 interfacial layer. *Nano Lett.* **11**, 2955-2961 (2011).
56. Calado, P. et al. Evidence for ion migration in hybrid perovskite solar cells with minimal hysteresis. *Nat. Commun.* **7**, 13831 (2016).
