# Supplementary material for: Down-selection of biomolecules to assemble “reverse micelle” with perovskites
Source: Nat Commun. 2024 Jan 26;15:772. doi: 10.1038/s41467-024-44881-4 (PMC10817902; doi:10.1038/s41467-024-44881-4)
Supplement: Supplementary file 3 — Solar Cells Reporting Summary [file 41467_2024_44881_MOESM3_ESM.pdf]

nature research

Corresponding author(s): Kai Wang

Solar Cells Reporting Summary

Nature Research wishes to improve the reproducibility of the work that we publish. This form is intended for publication with all accepted papers reporting the characterization of photovoltaic devices and provides structure for consistency and transparency in reporting. Some list items might not apply to an individual manuscript, but all fields must be completed for clarity.

For further information on Nature Research policies, including our data availability policy, see Authors & Referees.

Experimental design

Please check: are the following details reported in the manuscript?

1. Dimensions

Item	Yes/No	Details
Area of the tested solar cells	☒ Yes ☐ No	Aperture areas of 0.088, 3.76, 23.5 and 70 cm2 were used in this work, see in Methods.
Method used to determine the device area	☒ Yes ☐ No	Black metal aperture masks were used during the J-V measurements for solar cells with dimensions calibrated by optical microscopy, see in Methods.

2. Current-voltage characterization

Item	Yes/No	Details
Current density-voltage (J-V) plots in both forward and backward direction	☒ Yes ☐ No	See in Supplementary Figure 21f.
Voltage scan conditions<br>*For instance: scan direction, speed, dwell times*	☒ Yes ☐ No	See in Methods.
Test environment<br>*For instance: characterization temperature, in air or in glove box*	☒ Yes ☐ No	Device performance was measurement in ambient air under room temperature, see in Methods.
Protocol for preconditioning of the device before its characterization	☐ Yes ☒ No	No preconditioning was used in this work.
Stability of the J-V characteristic<br>*Verified with time evolution of the maximum power point or with the photocurrent at maximum power point; see ref. 7 for details.*	☒ Yes ☐ No	See in Supplementary Figure 25.

3. Hysteresis or any other unusual behaviour

Item	Yes/No	Details
Description of the unusual behaviour observed during the characterization	☒ Yes ☐ No	Minor hysteresis was observed for devices in this work (see in Supplementary Figure 21f).
Related experimental data	☒ Yes ☐ No	Forward and backward J-V scans were provided, see in Supplementary Figure 21f.

4. Efficiency

Item	Yes/No	Details
External quantum efficiency (EQE) or incident photons to current efficiency (IPCE)	☐ Yes ☒ No	MPPT and steady-state photo-current tests were provided to verify the photo-current value.
A comparison between the integrated response under the standard reference spectrum and the response measure under the simulator	☐ Yes ☒ No	As above.
For tandem solar cells, the bias illumination and bias voltage used for each subcell	☐ Yes ☒ No	Not relevant to this work.

5. Calibration

Item	Yes/No	Details
Light source and reference cell or sensor used for the characterization	☒ Yes ☐ No	450 W Xenon lamp (Oriel Sol 2A Class ABA) calibrated by a reference silicon cell covered by KG5 filter glass, see in Methods.
Confirmation that the reference cell was calibrated and certified	☒ Yes ☐ No	The reference cell was calibrated by Newport, see in Methods.

Calculation of spectral mismatch between the reference cell and the devices under test

☐ Yes
☒ No

We use KG5 filter glass of reference cell and M=1 for all the devices in this work.

6. Mask/aperture

Size of the mask/aperture used during testing

☒ Yes
☐ No

Metal aperture masks with areas of 0.088, 3.76, 23.5 and 70 cm2 were used for testing.

Variation of the measured short-circuit current density with the mask/aperture area

☐ Yes
☒ No

We measured all devices with masks.

7. Performance certification

Identity of the independent certification laboratory that confirmed the photovoltaic performance

☐ Yes
☒ No

This work focuses on conceptual advance in scientific understanding.

A copy of any certificate(s)
Provide in Supplementary Information

☐ Yes
☒ No

As above.

8. Statistics

Number of solar cells tested

☒ Yes
☐ No

20 devices under each different condition, see in Figure 3a and Supplementary Figure 9.

Statistical analysis of the device performance

☒ Yes
☐ No

See in Figure 3a and Supplementary Figure 9.

9. Long-term stability analysis

Type of analysis, bias conditions and environmental conditions
For instance: illumination type, temperature, atmosphere humidity, encapsulation method, preconditioning temperature

☒ Yes
☐ No

Shelf-stability measurement: devices under ambient air with encapsulation, see in Supplementary Figure 25a; MPPT: devices under continuous 1-sun illumination with encapsulation, see in Supplementary Figure 25c.
